# Accelerated burn wound healing with photobiomodulation therapy involves activation of endogenous latent TGF-β1

**DOI:** 10.1038/s41598-021-92650-w

**Published:** 2021-06-28

**Authors:** Imran Khan, Saeed Ur Rahman, Elieza Tang, Karl Engel, Bradford Hall, Ashok B. Kulkarni, Praveen R. Arany

**Affiliations:** 1 National Institute of Dental and Craniofacial Research, Bethesda, MD 20892 USA; 2 Oral Biology and Biomedical Engineering, University at Buffalo, 3435 Main Street, B36A Foster Hall, Buffalo, NY 14214 USA; 3 Present Address: National Cancer Institute, Bethesda, 20892 USA; 4 Present Address: Biotechnology Institute, Islamabad, Pakistan

**Keywords:** Biotechnology, Molecular biology, Molecular medicine, Optics and photonics

## Abstract

The severity of tissue injury in burn wounds from associated inflammatory and immune sequelae presents a significant clinical management challenge. Among various biophysical wound management approaches, low dose biophotonics treatments, termed Photobiomodulation (PBM) therapy, has gained recent attention. One of the PBM molecular mechanisms of PBM treatments involves photoactivation of latent TGF-β1 that is capable of promoting tissue healing and regeneration. This work examined the efficacy of PBM treatments in a full-thickness burn wound healing in C57BL/6 mice. We first optimized the PBM protocol by monitoring tissue surface temperature and histology. We noted this dynamic irradiance surface temperature-monitored PBM protocol improved burn wound healing in mice with elevated TGF-β signaling (phospho-Smad2) and reduced inflammation-associated gene expression. Next, we investigated the roles of individual cell types involved in burn wound healing following PBM treatments and noted discrete effects on epithelieum, fibroblasts, and macrophage functions. These responses appear to be mediated via both TGF-β dependent and independent signaling pathways. Finally, to investigate specific contributions of TGF-β1 signaling in these PBM-burn wound healing, we utilized a chimeric TGF-β1/β3 knock-in (TGF-β1^Lβ3/Lβ3^) mice. PBM treatments failed to activate the chimeric TGF-β1^Lβ3/Lβ3^ complex and failed to improve burn wound healing in these mice. These results suggest activation of endogenous latent TGF-β1 following PBM treatments plays a key role in burn wound healing. These mechanistic insights can improve the safety and efficacy of clinical translation of PBM treatments for tissue healing and regeneration.

## Introduction

Burn injuries are estimated to affect over 6 million people per annum worldwide^1^. These injuries cause significant morbidity (infections and scarring) and mortality associated with burn injuries. Aggressive clinical management guidelines have been developed based on the severity of burns, such as total body surface area, depth, and co-morbidities^2,3^. These strategies focus explicitly on the fundamental burn injury pathophysiology that evokes a range of thermal and cellular stress damage responses along with a prominent inflammatory sequela^4,5^. These include immediate thermal neutralization of burn damage, fluid resuscitation and nutrition, removal of necrotic tissue (escharotomy or fasciotomy), and rigorous general wound care principles^3,6,7^. Several non-invasive, biophysical modalities have been explored for burn wound therapies, best exemplified by hyperbaric oxygen (HBO) and negative pressure therapy^8,9^. However, the use of low-dose biophotonics therapy, termed Photobiomodulation (PBM) therapy, has gained much recent attention^10^.

Among its earliest clinical use, PBM was noted to effectively promote surgical wound closure^11^. Since then, the clinical benefits of PBM treatments have been extended in human clinical trials to various types of wounds such as diabetic, venous, pressure, and burns, among others^12^. PBM treatments have specifically shown discrete clinical benefits in effective management of burn wounds^13–24^. Various PBM parameters have been examined in these studies including wavelengths (ranging from 660 nm to near-infrared 904 nm), pulsing (0–80 Hz) and doses (2–25 J/cm^2^). Several systematic reviews and meta-analyses have rigorously established evidence for the clinical effectiveness of PBM treatments in mitigating inflammation and promoting wound healing^25–27^. A major recent milestone was the recommendation by the Multinational Association for Supportive Care in Cancer (MASCC) for the use of PBM treatments as a routine, standard-of-care treatment to manage oncotherapy-associated oral mucositis^28,29^.

While the precise responses mediating PBM-induced promotion of wound healing remains to be fully elucidated, there has been significant recent progress in our understanding of several molecular mechanisms of PBM^30,31^. These studies have focused on the effects of photosensitive endogenous targets such as intracellular (mitochondrial Cytochrome C oxidase), cell membrane receptors (TRPV1, Opsin 3 and 4), and extracellular complexes (TGF-β1)^32^. Among them, TGF-β is a multifunctional cytokine known to perform various cellular functions, including cell growth and differentiation, immunosuppression, angiogenesis, and wound healing^33,34^. Photoactivation of the latent TGF-β1 isoform, and not TGF-β2 or TGF-β3, is mediated via a specific methionine (position 253 on TGF-β1 latency associated peptide)^35,36^. The present study sought to examine the role of PBM-activated TGF-β1 in burn wound healing on the individual cellular responses and the role of inflammation by utilizing in vitro and in vivo animal models.

## Results

### Optimizing PBM treatment protocol to improve burn wound healing

**Figure 1:**
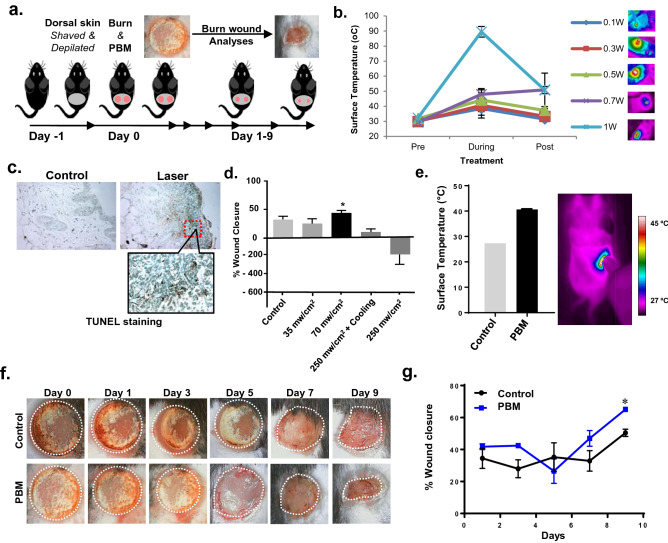
PBM treatment improves burn wound healing. (**a**) Representative diagram showing scheme of wound healing experiment. On the 1st day, the dorsal skin of 5-week-old C57BL/6NCr male mice was shaved, and on the 2nd day, two burn wounds were created, followed by PBM treatments (Day 0) with an 810 nm CW diode laser. Burn wounds were photographed every other day up to day 9; (**b**) Tissue surface temperature was monitored during laser treatments with increasing irradiances with a thermal camera; (**c**) Tissue damage was assessed in these tissues with TUNEL staining indicating phototoxicity at skin temperature above 45 °C; (**d**) Burn wound healing following PBM treatments at increasing doses and concomitant surface cooling, results are expressed as means and SDs representative of two independent experiments, significance was determined using non-parametric Student's *t*-test with *p-value < 0.05; (**e**) Optimal PBM dose treatments ensured skin surface temperature was > 45 °C using a dynamic irradiance protocol assessed with thermal imaging (left) and quantitation (right); (**f**) PBM treatments on burn wounds were photographed every other day for up to 9 days and compared to untreated controls; (**g**) Wound areas were digitally quantitated, results are expressed as means and SDs that is representative of five independent experiments, significance was determined using non-parametric Student's *T*-test with n = 8, *p-value < 0.05.

In this study, we utilized a modified burn wound model to generate consistent third-degree burns to evaluate the efficacy of PBM treatments (Fig. 1a)^37^. A near-infrared laser (810 nm, CW) was used to perform PBM treatments on shaved and depilated dorsum skin in C57/BL6 mice. We first wanted to establish an effective PBM treatment protocol as lasers can potentially cause inadvertent tissue thermal damage and exacerbate burn injury. We recently described the utility of monitoring tissue surface temperature during laser treatments using in vitro and in vivo models^38^. Hence, we first utilized an in vitro model to examine PBM dose-reciprocity (fluence, J/cm^2^) of both temporal (treatment time, sec) and laser treatment surface irradiance (mW/cm^2^)^39^. We observed that increasing laser irradiances results in significant damage that could be compensated by reducing treatment time to ensure surface temperature was maintained below 45 °C (Fig. S1a, b). This laser dose–response was confirmed in mice skin by monitoring surface temperature and examining clinical and histological tissue damage with TUNEL staining (Fig. 1b, c). Interestingly, reducing tissue temperature by surface cooling during laser treatments was able to partially reverse these detrimental effects in a monolayer cell wound 'scratch' assay in vitro (Fig. S1c). However, concomitant cooling during laser treatments was not effective at significantly improving wound closure in vivo (Fig. 1d). Based on these results, we established an optimal PBM protocol to promote wound healing with a dynamic (switching the laser on/off) laser irradiance at 70 mW/cm^2^ for 5 min for a total dose of 21 J/cm^2^ to maintain tissue surface temperature below 45 °C (Fig. 1e)^38^. Using this PBM treatment protocol, we examined burn wound healing responses over 9 days, and percent wound closure was assessed. PBM treated burn wounds had significantly improved burn wound healing compared to controls (*p* < 0.05) (Fig. 1f, g). These observations indicate that a non-invasive, low-dose biophotonics treatment could be effectively used in burn wound management.

### Wound epithelial responses in PBM-accelerated burn wound healing

**Figure 2:**
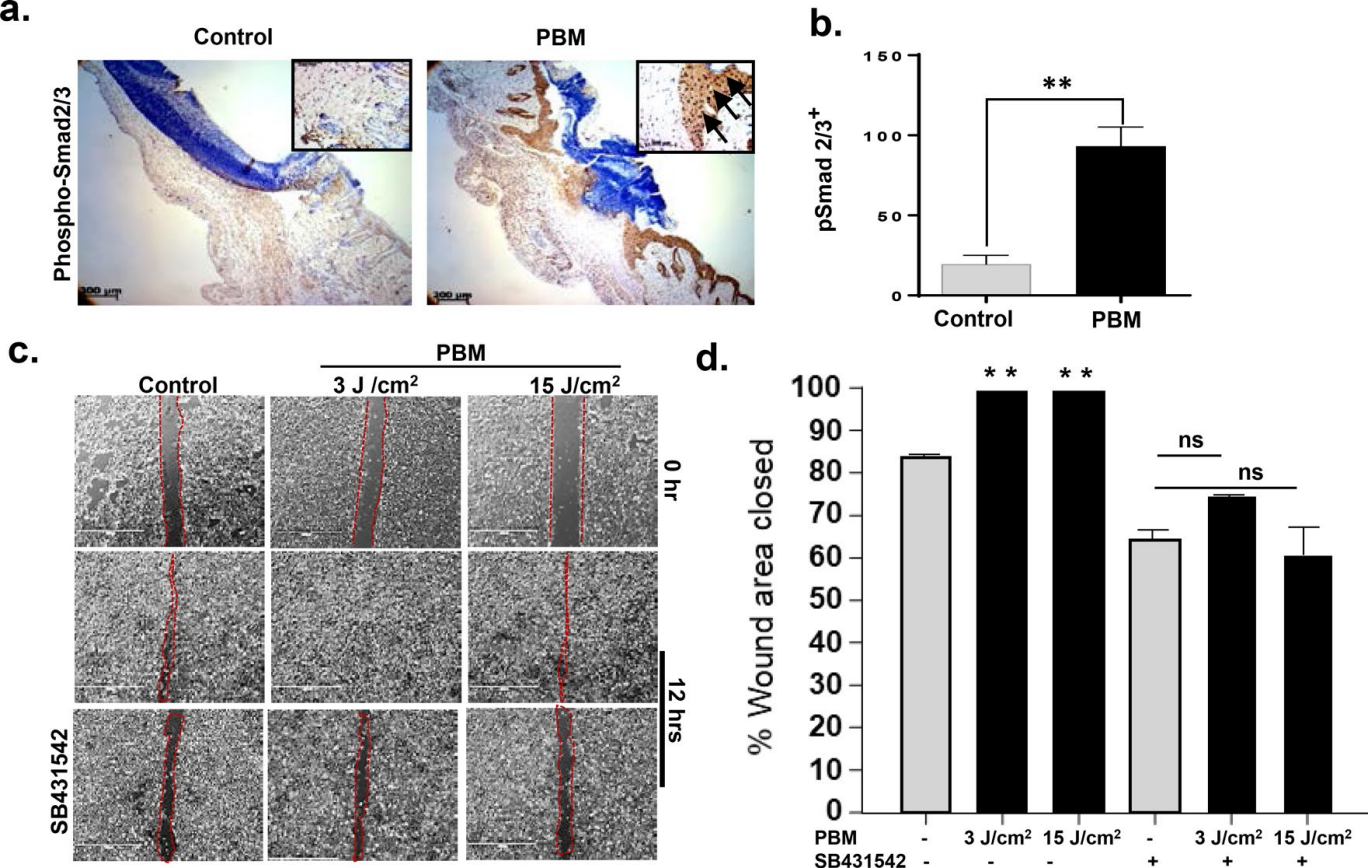
PBM activated TGF-β signaling promotes burn wound epithelial migration. (**a**) Burn wound tissues were immunostained for p-Smad2 at day 9; (**b**) Digital quantitation of immunohistochemical staining from mice sections, means and SDs are shown (n = 8, *p < 0.05, unpaired Student's *T*-Test); (**c**) Human dermal keratinocytes, HaCaT cells were plated in a 6-well tissue culture plate and were allowed to form confluent cultures for 24 h, and a scratch wound was created. PBM treatments at different doses with or without SB431542 inhibitor was performed, and images were captured with a digital microscope at 12 h; (**d**) Images were quantitated using T scratch software, and % area closed are shown as means and SDs that is representative of two independent experiments performed with replicates, significance was determined using one-way ANOVA among different treatments using the Tukey's multiple comparisons test indicated as **p < 0.005, *p < 0.05 and *n.s.* not significant.

Wound epithelization is a key aspect of successful healing orchestrated by a complex interplay of keratinocytes and underlying stroma involving several growth factors, including TGF-βs^40^. TGF-βs are secreted as latent complexes, and physicochemical activation is a key regulatory step in its pathophysiological roles^41^. Photoactivation of latent TGF-β1 is mediated by a redox-sensitive methionine (position 253 on latency-associated peptide) and has been shown to promote tissue healing and regeneration^35,36^. However, TGF-β has multifaceted roles in wound healing based on dose, timing, and context (cell lineages and crosstalk) specific responses^42^. Hence, we investigated lineage-specific responses to PBM-activated TGF-β signaling. We examined skin keratinocyte responses in PBM-treated and control burn wounds. At 9 days post-wounding, activation of TGF-β1 signaling was evident by increased nuclear localization of phospho-Smad2/3 in wound epithelial in PBM-treated wounds (*p* < 0.05) (Fig. 2a, b). Staining for TGF-β in serial sections demonstrated a slight increase, but it was not statistically significant (*p* > 0.05) (Fig. S2a). Together, these results suggest that PBM-activated endogenous TGF-β signaling in the epithelial cells may contribute to improved burn wound healing observed.

TGF-βs have been shown to promote proliferation and migration in a broad range of cell types from discrete anatomical sites^43–45^. We recently investigated the ability of the near-infrared laser to induce proliferation and enhance colony-forming units in epithelial keratinocytes^46,47^. These responses may contribute to the improved burn wound healing observed in this study. We examined the ability of PBM treatments to promote keratinocyte migration in vitro using both dermal and oral keratinocytes. We noted PBM treatments at 3 J/cm^2^ accelerated epithelial migration in both cell lines in the wound healing assay (Fig. 2c, d). To ascertain the roles of TGF-β signaling, pre-incubation with a TGF-βRI (Alk5) inhibitor, SB431542, was performed. Pretreatments with the inhibitor abrogated the observed accelerated epithelial migration (*p* < 0.05) (Fig. 2c, d). As oral healing occurs more rapidly with minimal scarring, we also examined PBM treatments on an oral keratinocyte cells that demonstrated a similar response (Fig. S2b, c)^48^. Together, these observations suggest PBM-activated TGF-β signaling in epithelial cells can contribute to improved wound closure.

### PBM-activated TGF-β1 induces myofibroblast phenotype

**Figure 3:**
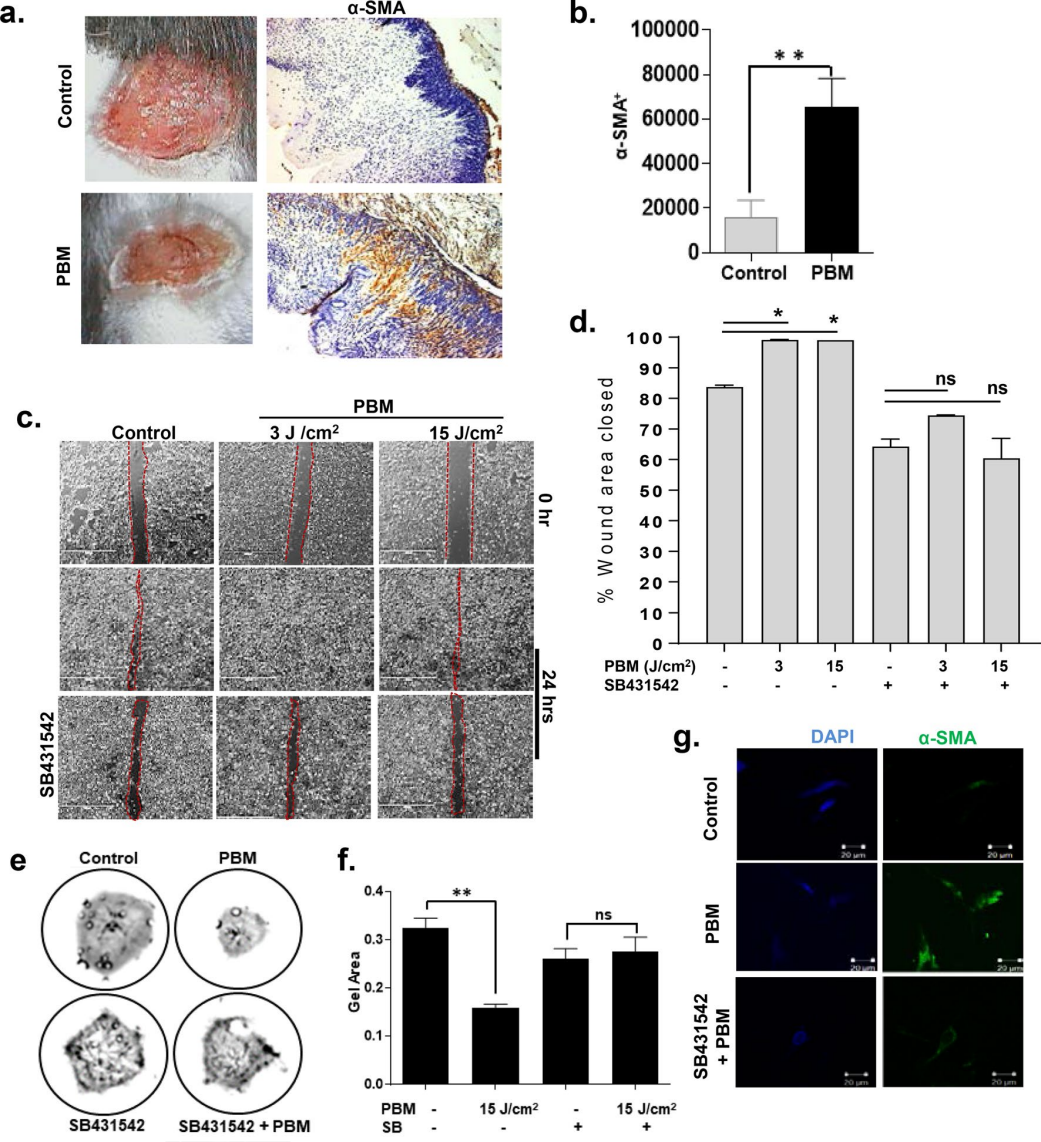
PBM activated myofibroblasts in burn wounds promotes wound contraction. (**a**) PBM treated mice were sacrificed, and wound areas were immunostained for α-SMA on day 9; (**b**) Digital quantitation of immunohistochemical staining from mice sections, means and SDs are shown (n = 8, unpaired *T*-Test, **p < 0.005); (**c**) Human dermal fibroblast cells were plated in a 6-well tissue culture plate and were allowed to form confluent cultures for 24 h, and a scratch wound was created. PBM treatments at different doses with or without SB431542 inhibitor was performed, and images were captured with a digital microscope at 12 h; (**d**) Images were quantitated using T scratch software, and % area closed are shown as means and SDs that is representative of two independent experiments performed with replicates, significance was determined using one-way ANOVA among different treatments using the Tukey's multiple comparisons test indicated as *p < 0.05 and *n.s.* not significant; (**e**) Collagen gel contraction assays were performed with dermal fibroblast cells cast in collagen gels plated in 24-well culture dishes. PBM treatments were performed at various doses with or without prior incubation with SB431542, and gels were then photographed after 24 h; (**f**) Gel areas were plotted, and data is shown as means and SDs that is representative of two independent experiments performed with replicates, statistical significance was determined with one-way ANOVA among different treatments using the Tukey's multiple comparisons tests, **p < 0.005; (**g**) Gels were fixed and immunostained for αSMA, and representative fluorescence images are shown.

A primary role for burn wound fibroblasts is acquiring a myofibroblast phenotype^49,50^. While transient expression of αSMA is necessary for enabling myofibroblast-mediated matric synthesis and wound contraction during normal healing, the persistence of these cells has been correlated with burn wound scarring and contractures^51,52^. A prior report had noted the ability of low dose laser treatment to increase myofibroblast transformation in vitro and in vivo*,* as evidenced by microfibrillar ultrastructural changes^53^. However, the precise mechanism mediating this process has not been elucidated. The healing burn wounds in the PBM treated group in our study appeared to have a puckered, oval shape (decreased short axis) that suggested prominent wound contraction was operative via a purse-string mechanism described previously^54^. To examine the role of PBM-induced myofibroblasts in these responses, we performed immunostaining for α-SMA in our burn healing tissues. We observed intense α-SMA expression in PBM-treated burn wounds compared to non-treated wounds (*p* < 0.05) (Fig. 3a, b). While the loose skin in mice undergoes significant wound contraction, specific models using splints or burn healing in other tight-skin species (porcine) could help further investigate effects of PBM on wound contraction more precisely^55,56^. These results indicate PBM-activated TGF-β signaling could actively mediate burn wound closure via transient myofibroblast transformation.

To investigate this further, we examined fibroblast proliferation, migration, and myofibroblast transformation following PBM treatments in vitro. In a prior study, we have observed PBM treatments affected epithelial and fibroblast survival differentially, with the latter cells being less sensitive^46^. PBM treatments prominently induce reactive oxygen species upstream of TGF-β signaling^57,58^. The increased PBM dose required for fibroblast responsiveness can be attributed to a higher antioxidant enzyme such as Catalase, Glutathione Peroxidase, or Superoxide dismutase^59,60^. Consistent with this observation, we noted PBM treatments were capable of significantly enhancing fibroblast migration (*p* < 0.05) at a higher PBM dose (15 J/cm^2^) (Fig. S3a–c). We further validated these effects are mediated via PBM-activated TGF-β by pre-incubation with SB431542 that abolished the pro-migratory responses in fibroblasts (*p* < 0.05) (Fig. 3c, d). Next, we examined the effect of PBM treatments on fibroblast-populated collagen lattices. PBM treatments at 15 J/cm^2^ induced α-SMA expression and induced gel contraction that was mediated via TGF-β signaling (Fig. 3e–g and Fig. S3d, e). These results together indicate PBM-activated TGF-β1 plays a key role in fibroblast-mediated burn wound healing.

### Role of macrophages in PBM treatments of burn wounds

**Figure 4:**
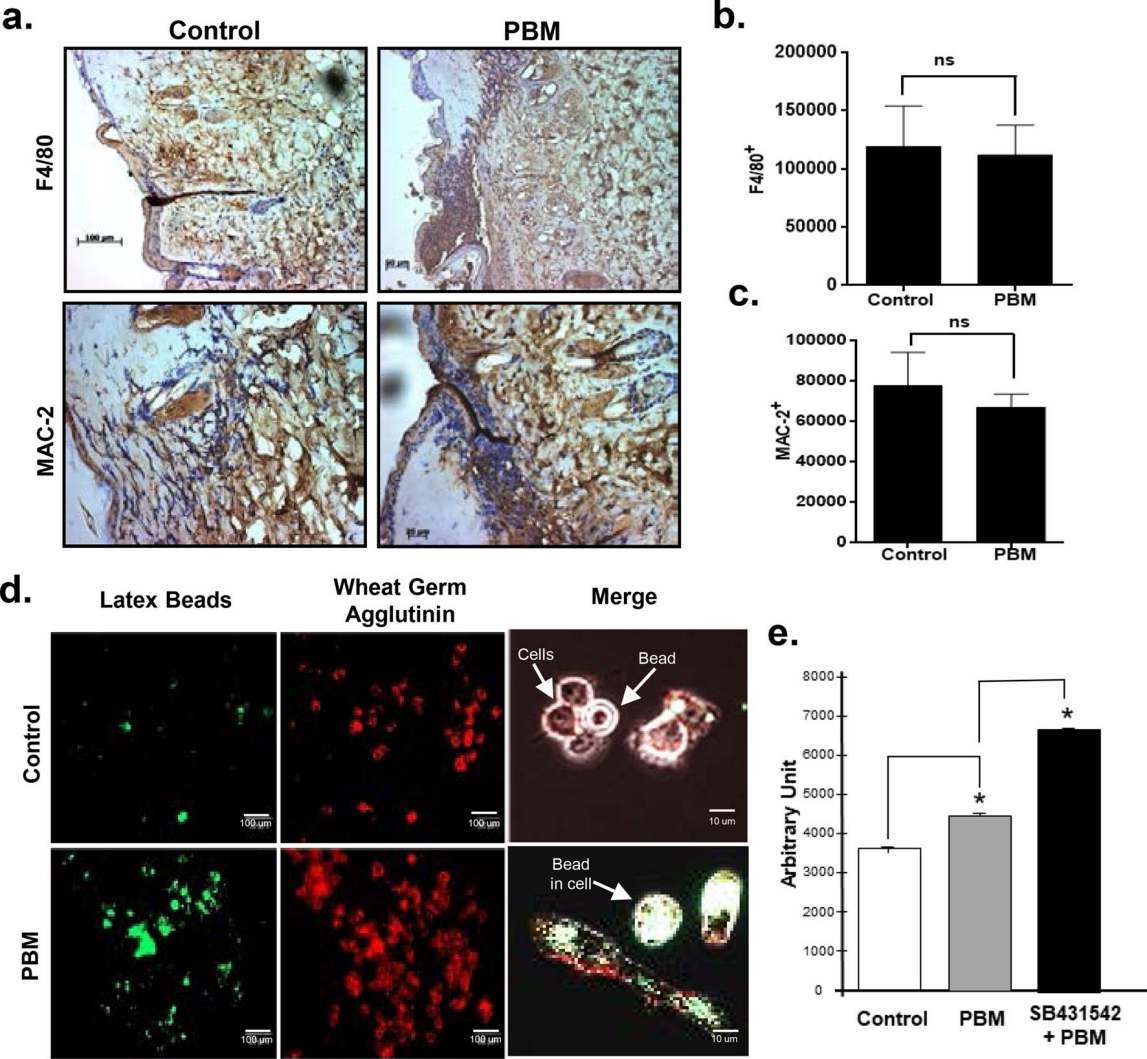
PBM treatments increased macrophage phagocytic activity. (**a**) Burn wound tissues from untreated and PBM treated mice at 24 h post-injury were immunostained for the macrophage markers F4/80 and Mac-2; (**b**) Quantitation of F4/80 immunohistochemical staining from mice sections is represented as means and SDs (n = 8, unpaired Student's *T*-Test, *n.s.* not significant); (**c**) Quantitation of Mac-2 immunohistochemical staining is shown as means and SDs are shown (n = 8, unpaired Student's *T*-Test, *n.s.* not significant); (**d**) Macrophage cell line RAW 264.7 cells were seeded in 96 well plates and incubated with FITC-labelled latex beads to examine phagocytosis. Cell membranes were counterstained with Texas Red-conjugated Wheat Germ Agglutinin and imaged using a fluorescence microscope; (**e**). Fluorescent intensity was quantitated using NIH ImageJ, and data are presented as means and SDs that are representative of two independent experiments performed with replicates; statistical significance was determined with one-way ANOVA among different treatments using the Tukey's multiple comparisons test, *p < 0.05.

A major distinguishing characteristic of burn wounds from other types of wounds is the prominent inflammation due to severe, protracted thermal tissue damage. The normal sequelae of healing are dramatically delayed in burn wounds until the persistent underlying inflammation is resolved. We first examined if PBM treatments affected the major phagocytic cells, macrophages in these mice burn wounds. We observed no significant differences in PBM-treated burn wounds for F4/80 and Mac-2 immunostaining compared to untreated control burn wounds (Fig. 4a–c). While the numbers of macrophages did not appear to change significantly, these cells play crucial roles in burn wound healing by clearing debris and preventing infections^61,62^. Therefore, we next examined the effects of PBM treatments on macrophage functions in vitro using a macrophage cell line, Raw264.7. Varying PBM doses failed to induce a proliferative response in macrophages in both basal and lipopolysaccharide-stimulated scenarios, although recombinant TGF-β1 treatments were noted to induce cell proliferation at varying doses (Fig. S4a, b).

Chemotactic infiltration of macrophages plays an important role in wound clearance and initiating resolution^63^. In our studies, PBM treatments accelerated macrophage migration, as did recombinant TGF-β1 treatments (Fig. S4c–e). However, pre-incubation with SB431542 appeared to significantly improve PBM-induced macrophage migratory response (Fig. S4f.). Another key macrophage function is to clear tissue debris and infiltrating microbes by phagocytosis. We investigated the effects of PBM treatments on macrophage phagocytic activity using a latex bead assay. PBM treatments significantly increased the macrophage phagocytic activity (Fig. 4d). Interestingly, recombinant TGF-β1 significantly reduced phagocytic activity, while pretreatments with SB431542 appeared to synergistically improve this PBM response (Fig. 4d, e, and Fig. S4g, h). These results together appear to indicate that while PBM treatments are capable of improving macrophage migration and phagocytosis, these responses appear to be independent of TGF-β signaling.

### PBM effects on the inflammatory response in burn wounds

**Figure 5:**
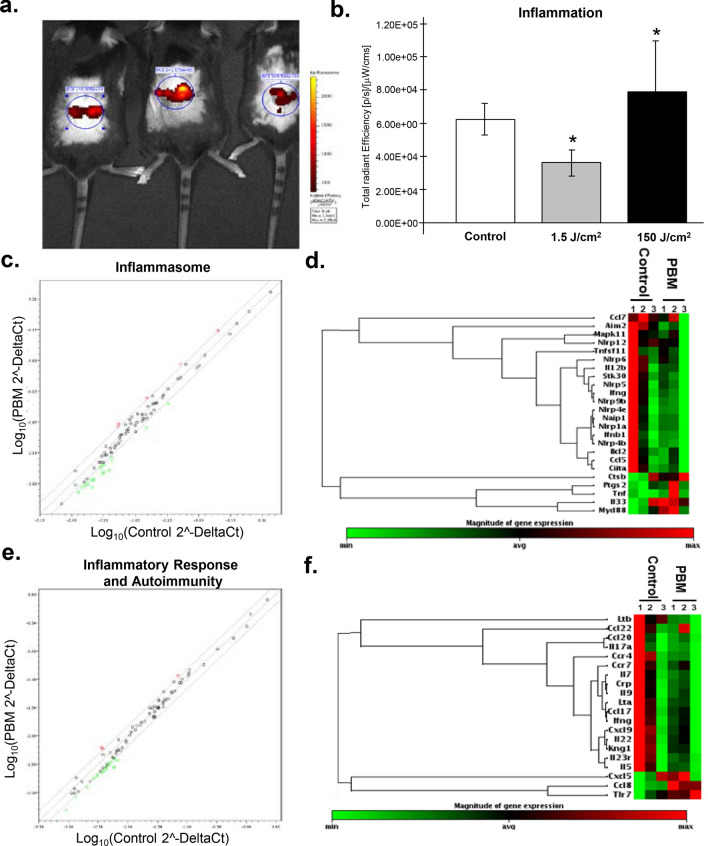
PBM downregulates inflammatory response in burn wounds. (**a**) Laser treatments were performed at varying doses and 24 h later mice were anesthetized and injected with the inflammation detection probe (XenoLight RediJect, 200 mg/kg, 100 μl per mice, i.p.) and live fluorescence imaging was performed; (**b**) Quantitation of fluorescence images using the Living Image software (Perkin-Elmer) and results are shown as means and SDs representative of two independent experiments with replicates, significance was determined using non-parametric Student's *t*-test with *p value < 0.05; (**c**) qPCR arrays were performed on wound tissues at 24 h of post-PBM treatments using inflammasome array and data is shown as a pair-wise comparison of gene expression in control versus PBM treated mice burn wounds with XY-scatterplot analysis of log base 2-transformed expression data, results represent two independent studies performed with triplicates; each dot represents a gene, with red dots showing genes denoting downregulated genes following PBM treatments with an FDR corrected *p* < 0.05, green dots represents upregulated genes with FDR < 0.05 and black dots are genes whose expression was similar between the two groups; (**d**) Hierarchical clustering of differentially regulated genes in untreated versus PBM treated burn wounds in the inflammasome array; (**e**) Similar qPCR arrays were performed on wound tissues at 24 h of post-PBM treatments using the inflammatory response and autoimmunity array and data is similarly presented as described above; (**f**) Hierarchical clustering of differentially regulated genes in the latter gene expression array.

There are well-established reports on the anti-inflammatory responses to PBM treatments, from lab studies to systematic reviews and human clinical trials^26,27,64–67^. Our prior cellular mechanistic in vitro studies utilized cell lines in an attempt to correlate the observed in vivo results in the mice studies. As the present PBM protocol utilized a single treatment immediately post-burn injury, we decided to investigate the direct responses of PBM treatments on the early inflammatory phase. Surgical lasers are effective surgical ablation tools capable of inducing thermal damage-mediated inflammation themselves. Therefore, we first inquired if the varying laser doses could modulate inflammation on the shaved and depilated (induces mild inflammation) unwounded mice skin using in vivo bioluminescence imaging. We noted the while high laser doses prominently increased inflammation due to thermal damage as expected, the low dose PBM laser treatments appeared to significantly reduce inflammation (Fig. 5a, b). Next, we sought to more broadly examine the reported anti-inflammatory signaling induced by PBM treatments. For this analysis, we assessed burn wound tissues at 24 h post-PBM treatments compared to untreated controls with two gene expression arrays involved in the inflammatory response. Of the 84 genes assessed, PBM treatments downregulated the expression of 18 genes (*Ccl7, Aim2, Mapk11, Nlrp12, Tnfsf11, Nlrp6, Il12b, Stk30, Nlrp5, Ifng, Nlrp9b, Nlrp4e, Naip1, Nlrp1a, Ifnb1, Nlrp4b, Bcl2, Ccl5,* and *Ciita*) and upregulated 5 genes (*Ctsb, Ptgs2, Tnf, Il33*, and *Myd88*) in inflammasome array (Fig. 5c, d, and Fig. S5a–c). Similarly, PBM treatments also downregulated the expression of 15 genes (*Ltb, Ccl22, Ccl20, Il17a, Ccr4, Ccr7, Il7, Crp, Il9, Lta, Ccl17, Ifng, Cxcl9, Il22, Kng1, Il23r*, and *Il5*) and upregulated 3 genes (*Cxcl5, Ccl8*, and *Tlr7*) among the 84 genes in the inflammatory response and autoimmunity array (Fig. 5e, f, and Fig. S5d–f). A KEGG pathway analysis of the differentially regulated genes highlighted the predominance of down-regulated genes that belonged to the cytokines-cytokines receptor interaction pathway (Fig. S5g). While a few of these genes appear to directly interact with TGF-β signaling, the overall assessment indicated down-regulation of the broader inflammatory pathways following PBM treatment (Fig. S5h, i). These results indicate that the direct anti-inflammatory responses to PBM treatments may involve both TGF-β dependent and independent pathways.

### PBM treatments fail to activate chimeric TGF-β1/3 knock-in complexes (TGF-β^L1β3/L1β3^)

**Figure 6:**
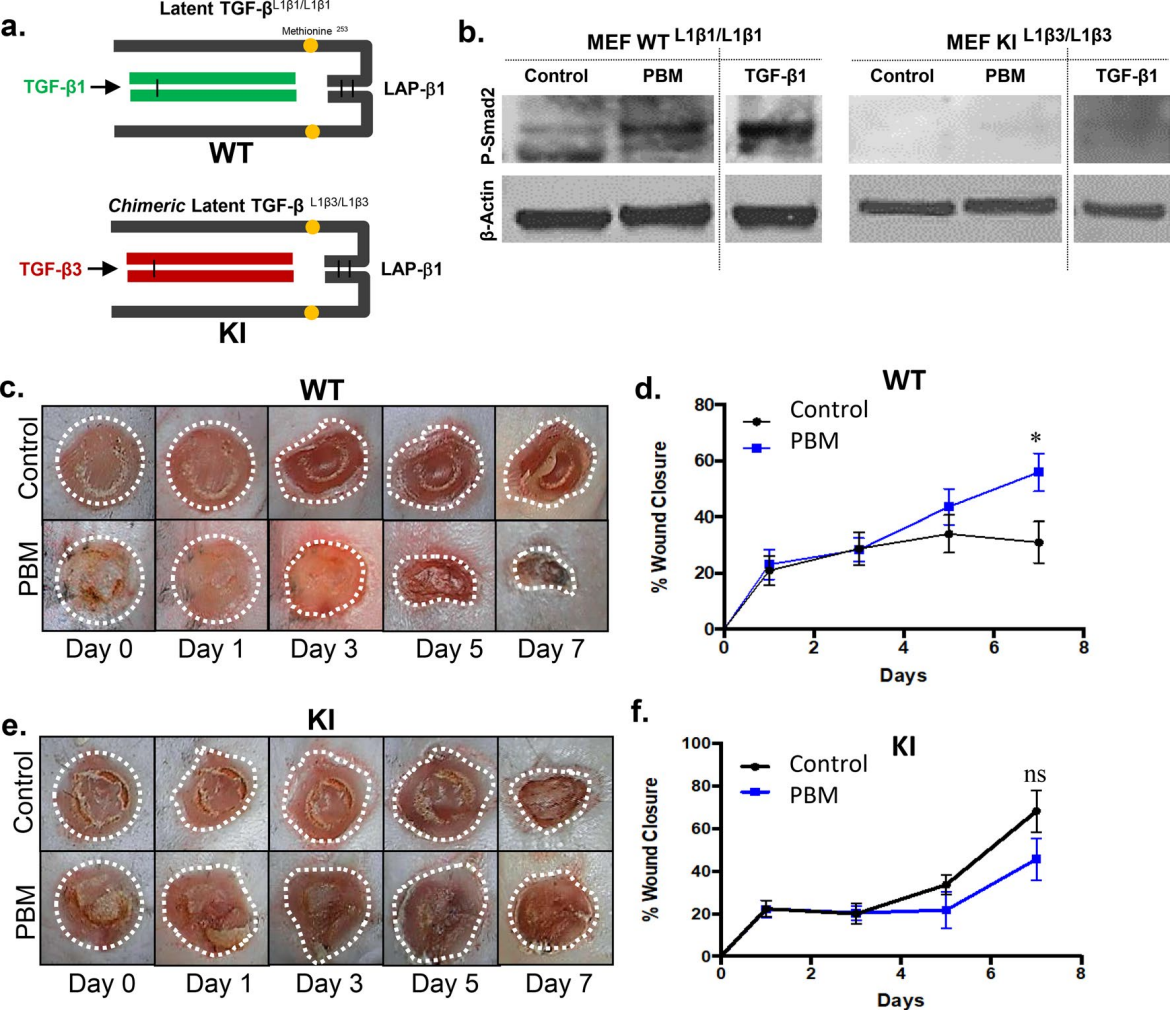
Lack of PBM-activated TGF-β fails to promote burn wounds in chimeric mice. (**a**) Schematic for wild type latent TGF-β^L1β1/L1β1^ and chimeric latent TGF-β^L1β3/L1β3^ knock-in mouse models; (**b**) MEFs obtained from both mice were PBM treated, lysed, and assessed for phospho-Smad2 by western blotting, recombinant TGF-β1 treatment was used as a positive control; (**c**) Burn wound healing and PBM treatments were performed in the WT mice, and digital images were captured for up to 7 days; (**d**) Wound areas were digitally quantitated, results are expressed as means and SDs representative of two independent experiments, n = 6, significance was determined using non-parametric Student's *t*-test with *p-value < 0.05; (**c**) Similarly, burn wound healing and PBM treatments were performed in the KI mice, and digital images were captured for up to 7 days; (**d**) Wound areas were digitally quantitated, results are expressed as means and SDs representative of two independent experiments, n = 6, significance was determined using non-parametric Student's *t*-test with, *n.s.* not significant.

The results from the gene expression and macrophage responses raised a critical question on if PBM-induced TGF-β signaling has a pivotal role in mediating the observed improvements in the burn wound healing model. To dissect this further, we chose to pursue a chimeric knock-in mouse model that has the TGF-β1 latency-associated peptide (LAP) with the mature β1 dimer (ligand) replaced with β3 (TGF-β1^L1β3/L1β3^) (Fig. 6a)^68^. Latent TGF-β complex can be dissociated by biophysical (extreme pH, heat, radiation) and biological (integrin-binding, thrombospondin-1) agents^69^. As noted previously, despite these broad range of activation avenues, activation of the latent TGF-β1 complex is the critical rate-limiting step in mediating its biological roles^41,69^. Our recent study had noted the ability of PBM-induced redox to activate latent TGF-β1 via the methionine residue at position 253 on the TGF-β1 LAP^35,70^. Moreover, TGF-β3 has been noted to generate more potent anti-inflammatory effects than TGF-β1^71,72^. As the LAP on other mammalian isoforms, TGF-β2 and TGF-β3, lack the redox-sensitive methionine, we speculated that PBM activation of the chimeric TGF-β1^L1β3/L1β3^ would generate more potent anti-inflammatory responses in burn wound healing.

We first generated mouse embryonic fibroblast (MEFs) cells derived from 15 days old pups of chimeric TGF-β1^Lβ3/Lβ3^ mice after tail genotyping to assess the cell-secreted complexes (Fig. S6a, b). Confluent MEFs cultures with their conditioned media containing either wild type latent TGF-β1 or the chimeric TGF-β1^Lβ3/Lβ3^ were subjected to PBM treatments. Surprisingly, western blotting for phospho-Smad2 demonstrated PBM treatment was incapable of activating the chimeric TGF-β^L1β3/L1β3^ in contrast to wild type latent TGF-β1 (Fig. 6b). We confirmed the chimeric TGF-β^L1β3/L1β3^ complex could be activated by routine chemical activation and recombinant TGF-β1 treatments induced phospho-Smad2 signaling in these MEFs (Fig. 6b)^68^. Thus, contrary to our expectation, we observed that the chimeric TGF-β^L1β3/L1β3^ could not be PBM activated. However, these observations also indicated that the chimeric TGF-β^L1β3/L1β3^ could be instead utilized to further examine the specific role of TGF-β1 contributions in PBM-treated burn wound healing.

### Laser failed to promote wound healing in chimeric TGF-β1/3 knock-in mice

We then subjected the chimeric TGF-β^L1β3/L1β3^ mice to burn wounds and performed PBM treatments, and compared them to wild type TGF-β^L1β1/L1β1^ and heterozygous chimeric knock-in (TGF-β^L1β1/L1β3^) mice. We noted the ability of PBM treatments to accelerate burn wound healing in wild type TGF-β^L1β1/L1β1^ mice, while a partial response was noted in the heterozygous chimeric TGF-β^L1β1/L1β3^ mice (Fig. 6c, d, and Fig. S6c, d). However, PBM treatments clearly failed to improve burn wound healing in the chimeric TGF-β^L1β3/L1β3^ mice (Fig. 6e, f, and Fig. S6e). We did not observe a significant difference in the inflammatory response in these mice with immunostaining for F4/80 and Mac-2, consistent with our prior studies in the C57BL6 mice (Fig. S6f, g). These results indicate TGF-β1 signaling has a central role in improved burn wound healing following PBM treatments.

## Discussion

Wound healing is a complex phenomenon with multiple cell types playing critical, discrete roles in the four overlapping phases of hemostasis, inflammation, proliferation, and remodeling^73,74^, Recruitment of inflammatory cells that include neutrophils, macrophages, and lymphocytes initiates the inflammation phase with its classical clinical signs of redness, warmth, swelling, and pain at the healing site^7^. Various growth factors play key roles in specific phases such as platelet-derived growth factor (PDGF), transforming growth factors-β (TGF-β), fibroblast growth factors (FGFs), and vascular endothelial growth factor (VEGF)^75^. TGF-β is a central wound healing cytokine with distinct effects on different cell types involved in wound healing responses, including cell migration and infiltration, angiogenesis, matrix synthesis, and remodeling^76–78^. The ability of PBM-activated TGF-β1 to promote wound healing prompted our interest in examining its efficacy in burn wound healing. This study highlighted a central role of TGF-β in mediating burn wound healing, specifically directed at the keratinocytes and fibroblast response. The ability to promote the reestablishment of the wound via both epithelial migration and wound bed contraction can significantly improve clinical wound outcomes. Wound closure is particularly relevant in burn wounds as the loss of barrier functions results in significant susceptibility to infections, fluid loss, and pain. Besides burn wounds, these cellular responses have important implications for broader clinical recommendations for the use of PBM treatments such as for chronic non-healing wounds and oncotherapy-associated mucositis^79^.

A dose and context-dependent responses are key characteristics to the complex roles of TGF-β signaling in health and disease^80^. Precise and tightly regulated TGF-β signaling is essential in ultimately determining therapeutic versus detrimental clinical outcomes^34^. In this study, we used a single in vivo PBM dose capable of directly activating latent TGF-β1 in the early burn healing phase that appeared to be adequate to promote healing. This was validated by the lack of healing observed in the chimeric TGF-β^L1β3L1β3^ mice. However, the partial improvement in healing evident in the chimeric heterozygous (TGF-β1^L1β1/Lβ3^) indicates a plausible dose-dependent response. Further, we observed varying PBM doses evoked discrete functional cellular responses in keratinocyte, fibroblasts and macrophage responses suggesting further improvements to both dose and timing of PBM protocols are feasible for specific wound phase management. A particular area of future investigation would focus on the macrophage subtypes (M1 versus M2) that have been demonstrated to be modulated by PBM treatments and are well known to contribute to the healing responses^81,82^. Evidences for TGF-β signaling in later healing stages could be attributed to the endogenous autocrine signaling. This is particularly significant clinically as persistent, dysregulated TGF-β signaling is known to play key roles in profibrotic, scarring phenotypes in burn wound healing^83,84^.

The ability to photoactivate TGF-β1 signaling provides an attractive endogenous optogenetic approach to dissect its central roles in human health and disease. PBM-mediated latent TGF-β1 activation is specifically mediated via a critical methionine at position 253 on the β1 LAP that is unique to this isoform, not TGF-β2 or TGF-β3^70^. We initially planned to utilize the chimeric β3 knock-in mice to extend this mechanism to TGF-β3, a potent immunomodulator and known to mediates scar-less wound healing in burn wounds^32,38^. In contrast to our expectation, the latent TGF-β1^L1β3/L1β3^ failed to photoactivate that could be attributed to the conformational restrictions of the chimeric complex. Therefore, it appears that we have serendipitously described a PBM mouse model that can be utilized to explore non-TGF-β1 responses. The results in this study clearly demonstrated several cellular responses, prominent anti-inflammatory responses and gene expression involved both TGF-β dependent and independent pathways (Fig. S7). The lack of healing in the PBM-treated chimeric TGF-β1^L1β3/L1β3^ burn wounds that has high basal and persistent post-burn inflammation suggests that TGF-β may play additional roles in fine tuning the inflammation and immune responses in burn healing. The complexity of TGF-β signaling with the presence of multiple ligands, receptors, accessory modulators, intracellular mediators involving Smads and other signaling intermediates particularly lends itself as an attractive central mediator orchestrating the healing responses^78,85^. Among various cellular mechanisms implicated in mediating therapeutic PBM effects in burn wound healing, the anti-inflammatory effects and regulation of mast cell degranulation have been noted to potentially play key roles^86,87^. Studies have noted roles for NFκB and PI3K-Akt signaling following PBM treatments that could be further investigated^88,89^. Nonetheless, the anti-inflammatory PBM mechanisms using genomics and proteomics could be further examined, perhaps in a less severe tissue injury model than the burn wounds represent^90^.

Tissue healing is teleologically important for survival, and a lack of wound healing generates significant morbidity and mortality^91,92^. The conventional mainstay of wound management involves surgical debridement, disinfection, and dressings. Newer approaches utilizing directed-energy, biophysical modalities such as microcurrents, ultrasounds, radiofrequency, and biophotonics have spurred much recent interest^93,94^. The use of biophotonics devices is based on specific photon-biological interactions and can assist in surgical debridement, disinfection (antimicrobial photodynamic therapy), and stimulation of host immune response and tissue healing or regeneration^95,96^. However, PBM therapy has been plagued with inconsistencies in clinical outcomes due to complexities in clinical dosing and delivery protocols^38^. Optimization of PBM clinical dosing and delivery is a key criteria for successful therapeutic clinical outcomes^32^. Inadvertent thermal dosing is perhaps a key contributor these clinical inconsistencies due to the innocuous nature of low-dose PBM treatments and the operator’s motivation to maximize therapeutic benefits. There are several successful strategies emphasizing the non-thermal nature of PBM protocols such as selecting a discrete wavelength such as visible light for superficial treatments and near-infrared for deeper targets, pulsing (increased thermal tissue relaxation), scanning delivery (small laser spot size) or large array (LED) patterns. Newer approaches such as surface tissue cooling, beam dose fractionation, up/down converting nanoparticles or photosensitizers and dynamic tissue temperature monitoring, as shown in this study, are also being effectively employed.

Our recent investigation focused on addressing the non-thermal nature of PBM and indicated maximal dose threshold can be monitored by assessing tissue temperature and ATF-4, an integrated stress mediator in the endoplasmic reticulum, in determining phototoxicity^38^. This study extended this observation in developing a precise burn wound healing protocol, ensuring no inadvertent additional thermal injury is generated with the near-infrared laser used for PBM treatments. There is now further evidence for the use of specific PBM wavelengths based on cellular chromophores such as flavins, opsins, aryl hydrocarbon receptors, and TRPV1 for targeted biological responses^97–100^. Future PBM protocols can be envisioned that will be directed at discrete wound phases such as hemostasis, inflammation, epithelial closure, matrix synthesis, wound contraction, angiogenesis, and remodeling. Moreover, this specific ability to target individual healing phases raises interesting questions on if PBM treatments using the innovative *dynamic irradiance surface temperature-monitored* PBM protocol in this study can prevent the cascading tissue damage in burn wound progression.

In conclusion, this study demonstrates the utility of PBM treatments in mitigating burn injury and provides the biological rationale for its clinical application in wound healing. Further clinical translation via human studies can provide a valuable tool for wound management.

## Materials and methods

### Cell lines and treatments

Dermal (HaCaT) and oral keratinocytes (NOKSI), fibroblasts (HOF), and macrophages (RAW 264.7) cells were maintained in DMEM (Sigma-Aldrich, USA) supplemented with 10% fetal bovine serum and 100 units/mL penicillin and 100 µg/mL streptomycin (All ThermoFisher). Cells were incubated at 37 °C in a humidified chamber with 5% CO_2_. For treatments, cells were serum-deprived for 24 h and were then treated (TGF-β1 2.5 ng/ml, 810 nm laser) for different time intervals. For treatment with SB431542 (10 uM, TGF-β RI inhibitor, Sigma-Aldrich,) cells were pre-treated for 30 min prior to treatments.

### Photobiomodulation treatments

We performed PBM treatments using an 810 nm, continuous wave GaAlAs laser (AMD lasers, USA) as described previously^38^. An infrared camera ICI7640 (Infrared Cameras Incorporation, USA) was used to measure surface temperature of skin. For the in vivo studies, the laser probe was setup 2 cm perpendicular to the mouse with a spot size of 2 cm in diameter and tissue surface irradiance was assessed with a power meter (Thor labs). The laser was used for various treatment time based on the melanin score and dynamically adjusted (laser switched on/off) to maintain specific surface temperature (45–55 °C) as monitored by the IR camera. For in vitro studies, treatments were performed at a given distance and tissue surface irradiance was assessed with a power meter (Thor labs).

### Mouse embryonic fibroblasts (MEFs)

Mouse Embryonic fibroblasts were utilized to assess cell-secreted latent TGF-β1 complexes. Female mice were sacrificed by cervical dislocation, and 15–17 day old fetuses were dissected in 100 mm tissue culture plates with PBS. For genotyping of the pubs, the tail was removed, and PCR was performed as described earlier^68^. After removal of internal organs, each carcass was subjected to a tail snip and then transferred to a six-well plate (one carcass per well) with 3 ml of 0.5% trypsin (Gibco), minced using scissors, and incubated for 20–30 min at 37 °C. Following incubation, trypsin was neutralized using 5 ml of DMEM containing 10% FBS, and the cell suspension was passed through a 40 µm cell strainer and centrifuged for 5 min at 1000 rpm. Pellets were resuspended in 1 ml complete media and plated in T25 flasks for sub-cultures. All MEFs were used within passage 7 for this study.

### Cell proliferation assay

Cells were seeded in 96 well plates with a density of 3 × 10^3^ cells/well. Following overnight incubation, cells were treated with Lipopolysaccharide (*E. coli* 0111: B4 LPS, 1 µg/ml), TGF-β1 (2.5 ng/ml), SB431542 (10 µM), or lasers (10–1000 mW/cm^2^). After 24 h, cells were incubated with 10% (v/v) AlamarBlue in complete media for 2 h at 37 °C. Relative fluorescence intensity was assessed (560/590 nm) with a microplate reader (i3Max, Molecular Devices).

### Cell migration assays

To examine the effects of PBM treatments on cellular migration, scratch wound healing 'scratch' assays were performed. Cells were plated at high density (1 × 10^5^ cells per well in 6 well plate), and the following day, confluent monolayer cells were scraped using a micropipette tip to generate a scratch wound, treatments were performed, and cells were photographed over time (12, 24 and 48 h) using a digital microscope (Olympus). Images were quantitated using T scratch software based on the differences in the open scratch (wound) area. For the RAW264.7 studies, cells were pre-treated with Lipopolysaccharide (*E. coli* 0111:B4 LPS, 1 µg/ml) prior to treatments.

### Collagen gel contraction assay

Three-dimensional collagen gels have been widely used in assessing the activation status of fibroblasts^101^. Collagen gel with fibroblast cells was cast with the following composition: 4 × DMEM solution containing sodium bicarbonate were mixed with water, Rat Tail Tendon Collagen (RTTC 2 mg/ml), and fibroblast cells suspension (2 × 10^6^). This collagen gel mixture was cast in a 24 well plate (500 µl per well) and was allowed to form a gel at 37 °C for 45 min. Following this, 500 µl of reconstituted DMEM was added to each well, and the gel was released with the help of a pipette tip or scapula. This was followed by the treatment with different treatments, and gels were then photographed at 24 h.

### Phagocytosis assay

RAW 264.7 cells were seeded in 96 well plates with a density of 1 × 10^4^ cells/well and incubated overnight. Latex beads-rabbit IgG-FITC (ThermoFisher) was added to the freshly prepared culture medium at a 1:200 dilution. Cells were treated and then incubated for a further 24 h. Cells were then stained with Texas Red-conjugated Wheat Germ Agglutinin (WGA) (ThermoFisher), washed with assay buffer twice, and examined using a fluorescence microscope (ZOE, BioRad). Fluorescent intensity was assessed with ImageJ (NIH) software.

### Immunocytochemistry

Immunocytochemistry was performed on collagen gel by first fixing and permeabilized using cold methanol for 5 min at − 20 °C. Following a brief PBS wash, 1% BSA was used to block non-specific staining for 1 h at room temperature. Then, gels were incubated with α-SMA primary antibody overnight (Abcam, 1:100) at 4 °C. The following day, collagen gels were washed in PBS and incubated with a secondary antibody (Anti-rabbit Alexa Fluor 488) for 1 h. Finally, gels were stained with DAPI containing antifade solution and visualized using a confocal scanning electron microscope (Carl-Zeiss).

### Immunohistochemistry (IHC)

All tissue sections were first routinely examined by Hematoxylin and Eosin (H&E) staining to outline the wound and healing areas. Serial sections were deparaffinized and hydrated, followed by antigen retrieval by boiling in 10 mM citrate buffer (pH 6.0) using a microwave oven for 3 min at the highest power setting. Sections were allowed to cool at room temperature, washed twice in PBS, and treated with 5% hydrogen peroxide in methanol for 5 min to block the endogenous peroxidase activity. Following a PBS wash, skimmed milk powder (5%) was used to block non-specific background staining for 1 h. Sections were then incubated overnight at 4 °C with primary antibodies, namely TGF-β1 (Promega, 1:100), phospho-Smad2/3 (Cell signaling technology, 1:50), α-SMA, F4/80 and Mac-2 (All Abcam, 1:100). This was followed by incubation with horseradish peroxidase detection system (Biogenex, USA) for 20 min each and DAB (Sigma-Aldrich, USA) as the chromogenic substrate for visualization. Sections were imaged on a light microscope with a digital camera (Nikon).

### Western blot analysis

Semi-confluent cultures of Mouse Embryonic Fibroblast cells (MEFs) were treated and were washed in PBS and lysed with RIPA lysis buffer containing protease inhibitor cocktail (both, Sigma Aldrich, USA). Total protein was determined using the BCA reagent (Pierce, USA). An equal amount of protein extracted from cells was resolved on 2–10% gradient SDS-PAGE gel, transferred to polyvinylidene difluoride (PVDF) membrane, and subjected to immunoblot analysis. To block non-specific binding sites, blots were incubated in blocking buffer (Licor) for 1 h followed by overnight incubation in primary antibodies phospho-Smad2/3 (Cell signaling technology, 1: 1000) and β-Actin (Cell Signaling Technology, 1: 10,000) at 4 °C in 1% BSA. Blots were washed in Tris-buffered saline with Tween (TBST) thrice (10 min each) followed by incubation with secondary antibody (Anti-rabbit, mouse or goat IRDye 800 or 680 CW, Licor, 1:10,000) for 1 h. Following TBST washes, bands were visualized using two-channel Odyssey Imaging Systems (Licor, USA) and quantitated using AlphaImager software (ProteinSimple, USA). Quantitation is presented compared to control (β-Actin).

### In vivo wound healing studies

All study protocols were approved by the Institutional Animal Care and Use Committee (IACUC), NIDCR/NIH. All experimental protocols were approved institutional guidelines committee and animal studies were carried out in compliance with the ARRIVE guidelines. Over 150 mice aged at 4–6 weeks were either procured from commercial vendor (Jackson Laboratory, USA) or inbred (transgenics). C57BL/6NCr or C57.FV/Tg (TGF-β1/β3 knock-in and WT littermate) were anesthetized with 2–5% isoflurane gas, and the dorsal skin fur was clipped and shaved (Wahl Clippers Corp.), followed by depilation (Nair, Church, and Dwight). The following day, two anatomically-discrete skin burn wounds were created on the dorsal side on the following day with a brass disc heated to 185°F (85 °C), stabilized for 2 min, and placed firmly on the skin for 10 s. PBM treatments were performed with a commercial clinical diode laser unit (AMD, 810 nm) used at a tissue surface irradiance (10 mW/cm^2^) for 5 min to deliver total energy of 3 J/cm^2^ using a *dynamic irradiance-surface temperature protocol* that prevents phototoxicity as described previously^38^. Further, the nature of NIR ensures underlying (up to 1 cm subdermal) and large spot size would treat viable tissues at burn wound margins effectively. Wounds were monitored, and calibrated digital photographs were assessed.

### RNA extraction and PCR arrays

Burn wounds from mice were collected in RLT buffer (containing β-ME), and tissues were homogenized and centrifuged for 3 min at 14,000 rpm at 4 °C. mRNA was isolated using RNeasy spin column (Qiagen), and cDNA was synthesized using 2 μg of RNA (cDNA synthesis kit, Applied Biosystems) as per manufacturer's protocols. RT^2^ Profiler PCR Arrays (PAMM-077ZC and PAMM-097ZC) were performed using 20 ng cDNA with RT^2^ SYBR Green PCR Master mix as per manufacturer's protocol (RT^2^ Profiler PCR Array, Qiagen, USA) using StepOnePlus Real-time PCR and data were analyzed using the online software http://pcrdataanalysis.sabiosciences.com/pcr/arrayanalysis.php.

### In vivo imaging for inflammation

Mice were anesthetized with 4% isoflurane via a calibrated vaporizer connected to a gas evacuation unit, dorsal fur was clipped and shaved (Wahl Clippers Corp.), followed by depilation (Nair, Church, and Dwight). Immediately following this procedure, mice were subjected to laser (810 nm, CW, 0.1 W or 1 W for 300 s) treatments and recovered. After 24 h, mice were anesthetized again and injected with the inflammation detection probe (XenoLight RediJect, PerkinElmer, 200 mg/kg, 100 μl per mice, i.p.) 5 min prior to imaging, placed in the IVIS Lumina XR imaging station, and fluorescence images were acquired and quantitated with the Living Image software (Perkin-Elmer).

### Statistical analysis

Data was organized in Excel (Microsoft) and analyzed in GraphPad Prism (IBM). All in vitro studies were repeated at least two or three times, with each study performed with replicates. In vivo mice, studies were repeated at least two to five times. Representative data are presented as means with standard deviation, and statistical significance was assessed with either Student's *T*-Test or one-way ANOVA with post-hoc Tukey for multiple comparisons.

## Supplementary Information

Supplementary Information 1.

Supplementary Information 2.

## Abbreviations

PBM: Photobiomodulation
TGF-β: Transforming Growth Factor-β
