# Author Correction: Accelerated burn wound healing with photobiomodulation therapy involves activation of endogenous latent TGF-β1

**DOI:** 10.1038/s41598-021-97271-x

**Published:** 2021-08-31

**Authors:** Imran Khan, Saeed Ur Rahman, Elieza Tang, Karl Engel, Bradford Hall, Ashok B. Kulkarni, Praveen R. Arany

**Affiliations:** 1 National Institute of Dental and Craniofacial Research, Bethesda, MD 20892 USA; 2 Oral Biology and Biomedical Engineering, University at Buffalo, 3435 Main Street, B36A Foster Hall, Buffalo, NY 14214 USA; 3 Present Address: National Cancer Institute, Bethesda, 20892 USA; 4 Present Address: Oral Biology, Institute of Basic Medical Sciences, Khyber Medical University, Peshawar, Pakistan

Correction to: *Scientific Reports* 10.1038/s41598-021-92650-w, published online 28 June 2021

The original version of this Article contained an error in Affiliation 4, which was incorrectly given as ‘Biotechnology Institute, Islamabad, Pakistan’. The correct affiliation is listed below:

Oral Biology, Institute of Basic Medical Sciences, Khyber Medical University, Peshawar, Pakistan

The original Article has been corrected.

