# Supplementary material for: Accelerated burn wound healing with photobiomodulation therapy involves activation of endogenous latent TGF-β1
Source: Sci Rep. 2021 Jun 28;11:13371. doi: 10.1038/s41598-021-92650-w (PMC8238984; doi:10.1038/s41598-021-92650-w)
Supplement: Supplementary file 1 — Supplementary Information 1. [file 41598_2021_92650_MOESM1_ESM.docx]

**Supplementary Information**

**Accelerated Burn Wound Healing with Photobiomodulation Therapy involves activation of endogenous latent TGF-β1**

Imran Khan^1, #^, Saeed Ur Rahman^2,$^, Elieza Tang^1^, Karl Engel^1^, Bradford Hall^1^, Ashok B Kulkarni^1,^ and Praveen R Arany^1,2^

1. National Institute of Dental and Craniofacial Research, Bethesda MD, 20892
2. Oral Biology and Biomedical Engineering, University at Buffalo, NY 14214

**SUPPLEMENTARY FIGURE LEGENDS**

**Supplementary Figure 1:** (**a**) A 810 nm CW diode laser was used at varying irradiances and treatment time on HaCaT cells in black-well plates, and surface temperature was assessed using a thermal camera; (**b**) Cells viability was assessed 24 hrs post-laser treatments using AlamarBlue assay demonstrating limited dose-reciprocity, the dotted line represents 50% cell viability considered a measure of discrete phototoxicity, data are presented as means & SDs; (**c**) Keratinocytes were plated at high density in a 6-well culture plate and allowed to form confluent cultures, and a wound 'scratch' assay was performed followed by PBM treatments at either room temperature (27^o^C) or at 4°C (Cryospray). Wound scratch area was quantitated at 12, 24, and 48 hrs using a digital microscope, and percent (%) area closed is plotted using the T-Scratch software, data is presented as means & SDs.

**Supplementary Figure 2:** (**a**) Burn wound tissues were immunostained for TGF-β at day 9 that were digitally quantitated, means & SDs are shown (n = 8, n.s. = not significant, unpaired Student's T-Test); (**b**) Human oral keratinocytes were plated in a 6-well tissue culture plate and were allowed to form confluent cultures for 24 hrs and a scratch wound was created. PBM treatments at different doses with or without SB431542 inhibitor was performed, and images were captured with a digital microscope at 12 hrs; (**c**) Images were quantitated using T scratch software, and % area closed are shown as means & SDs that is representative of two independent experiments performed with replicates, significance was determined using one-way ANOVA among different treatments using the Tukey's multiple comparisons test indicated as *p < 0.05, **p < 0.005, n.s. = not significant.

**Supplementary Figure 3:** (**a**) Human dermal fibroblast cells were plated in a 6-well tissue culture plate and were allowed to form confluent cultures overnight, and a scratch wound was created. PBM treatments at different doses were performed, and images were captured with a digital microscope at 24 and 48 hrs; Wound images were quantitated at (**b**) 24 hours and (**c**) 48 hours using T scratch software, and % area closed are shown as means & SDs that is representative of two independent experiments performed with replicates, significance was determined using one-way ANOVA among different treatments using the Tukey's multiple comparisons test indicated as * p < 0.05, n.s. = not significant; (**d**) Collagen gel contraction assays were performed with dermal fibroblast cells cast in collagen gels plated in 24-well culture dishes. PBM treatments were performed at various doses with or without prior incubation with SB431542, and gels were then photographed after 24 hrs; (**e**) Gels were fixed and immunostained for αSMA, and representative fluorescence images are shown.

**Supplementary Figure 4:** (**a**) Macrophage cell line RAW 264.7 cells were seeded in 96 well plates and treated with varying dose of recombinant TGF-β1 and cell proliferation was assessed with AlamarBlue assay at 24 hours, data is presented as means & SDs that is representative of two independent experiments performed with replicates, significance was determined using one-way ANOVA among different treatments using the Tukey's multiple comparisons test indicated as * p < 0.05; (**b**) RAW 264.7 cells were seeded in 96 well plates and varying doses of PBM treatments were performed in the absence or presence of lipopolysaccharide, data is presented as means & SDs that is representative of two independent experiments performed with replicates, significance was determined using one-way ANOVA among different treatments using the Tukey's multiple comparisons test; (**c**) RAW 264.7 were plated in a 6-well tissue culture plate and were allowed to form (loose) confluent cultures overnight and a scratch wound was created. TGF-β1 treatments at different doses were performed, and images were captured with a digital microscope at 24 hrs; (**d**) Wound images were quantitated at 24 hours using T scratch software, and % area closed are shown as means & SDs that is representative of two independent experiments performed with replicates, significance was determined using one-way ANOVA among different treatments using the Tukey's multiple comparisons test indicated as * p < 0.05; (**e**) To assess the effect of PBM treatments, RAW 264.7 were similarly plated in a 6-well tissue culture plate, allowed to form (loose) confluent cultures overnight and a scratch wound was created. PBM treatments with or without SB431542 was performed, and images were captured with a digital microscope at 24 hrs; (**f**) Wound images were quantitated at 24 hours using T scratch software, and % area closed are shown as means & SDs that is representative of two independent experiments performed with replicates, significance was determined using one-way ANOVA among different treatments using the Tukey's multiple comparisons test indicated as * p < 0.05; (**g**) Phagocytosis assay was performed with RAW 264.7 cells seeded in 96 well plates, incubated with FITC-labelled latex beads and treated with TGF-β1 in the absence or presence of SB431542, cell membranes were counterstained with Texas Red-conjugated Wheat Germ Agglutinin and imaged using a fluorescence microscope; (**e**). Fluorescent intensity was quantitated using NIH ImageJ, and data are presented as means & SDs that are representative of two independent experiments performed with replicates; statistical significance was determined with one-way ANOVA among different treatments using Tukey's multiple comparisons tests, * p < 0.05.

**Supplementary Figure 5:** (**a**) Burn wounds of 5-week-old C57BL/6NCr male mice were treated with PBM, and tissues were collected at 24 hrs post-laser treatment, and qPCR array for inflammasome was performed. Heat maps and hierarchical clustering of differentially regulated genes in untreated versus PBM treated burn wounds are shown from three animals per group performed in replicates; (**b**) Fold change of downregulated genes from Inflammasomes array in the PBM treated group is outlined; (**c**) Fold change of upregulated genes from Inflammasomes array in the PBM treated group is outlined; (**d**) Similar analyses were carried out with the qPCR array for Inflammatory Response & Autoimmunity pathway that has 86 discrete genes among them. Heat maps and hierarchical clustering of differentially regulated genes in untreated versus PBM treated burn wounds are shown from three animals per group performed in replicates; (**e**) Fold change of downregulated genes from Inflammatory Response & Autoimmunity array in the PBM treated group is outlined; (**f**) Fold change of upregulated genes from Inflammatory Response & Autoimmunity array in the PBM treated group is outlined; (**g**) Differentially regulated genes obtained from Inflammasomes and Inflammatory Response & Autoimmunity PCR arrays were fed into Kyoto Encyclopedia of Genes and Genomes (KEGG) pathway analysis software and image shows genes with green highlight indicating down-regulation, red indicating upregulation and black are non-regulated; The KEGG is a database resource that integrates genomic, chemical and systemic functional information developed by Kanehisa Laboratories (https://www.kegg.jp/kegg/kegg1.html). (**h**) The down-regulated gene lists from both array were uploaded to an online website GeneMANIA (http://genemania.org) mania for *mus musculus* that highlighted interactions have been collected from GEO, BioGRID, IRefIndex and I2D, as well as organism-specific functional genomics data sets. TGF-β is specifically highlighted in red; (**i**) Similarly, upregulated gene lists from both arrays were uploaded to Genemania for *mus musculus,* and the TGF-β pathway is highlighted in red.

**Supplementary Figure 6:** (**a**) Schematic outline isolation of Mouse Embryonic Fibroblasts (MEFs) from 15 days' pregnant wild type TGF-β^L1β1/ L1β1^ and chimeric TGF-β^L1β3/ L1β3^ knock-in mice; (**b**) Genotyping for wild type TGF-β^L1β1/ L1β1^ and TGF-β^L1β3/ L1β3^ alleles from isolated primary MEFs, the entire unprocessed blot is shown on the right (**c**) Burn wound healing and PBM treatments were performed at different doses in the wild type TGF-β^L1β1/ L1β1^ mice and digital images were captured for up to 7 days and quantitated, data is presented as means & SDs representative of two independent experiments , n = 5; (**d**) Similarly, burn wound healing and PBM treatments were performed at different doses in the heterozygous TGF-β^L1β1/ L1β3^ mice and digital images were captured for up to 7 days and quantitated, data is presented as means & SDs representative of two independent experiments , n = 4; (**e**) Finally, burn wound healing and PBM treatments were performed at different doses in the wild type TGF-β^L1β1/ L1β1^ mice and digital images were captured for up to 7 days and quantitated, data is presented as means & SDs representative of two independent experiments , n = 4; (**f**) Wound tissues were collected and were stained for macrophage markers F4/80 and quantitation is presented as means & SDs, statistical significance was determined using one-way ANOVA among different treatments using the Tukey's multiple comparisons test, **p < 0.005, n.s. = not significant; (**g**) Wound tissues were collected and were stained for macrophage markers Mac-2 and quantitation is presented as means & SDs, statistical significance was determined using one-way ANOVA among different treatments using the Tukey's multiple comparisons test, *p < 0.05, n.s. = not significant; (**h**) Western blots for Figure 6B for PhosphoSmad2 and βActin, complete blots with areas cropped for presentation highlighted.

**Supplementary Figure 7:** Schematic outline of Photobiomodulation (PBM) therapy that generates reactive oxygen species and activates both TGF-β dependent and independent pathways that modulate individual cell type responses. The prominent anti-inflammatory response evoked by PBM treatments also appears to involve both pathways. The tabular outline lists the individual cellular responses to PBM and TGF-β1treatments as well as the loss of function studies outlining the role of PBM-activated TGF-β signaling in these responses. This summary is based on results from this study and prior reports from our group cited in this work.
