# Supplementary material for: Accelerated burn wound healing with photobiomodulation therapy involves activation of endogenous latent TGF-β1
Source: Sci Rep. 2021 Jun 28;11:13371. doi: 10.1038/s41598-021-92650-w (PMC8238984; doi:10.1038/s41598-021-92650-w)
Supplement: Supplementary file 2 — Supplementary Information 2. [file 41598_2021_92650_MOESM2_ESM.ppt]

## Slide 1
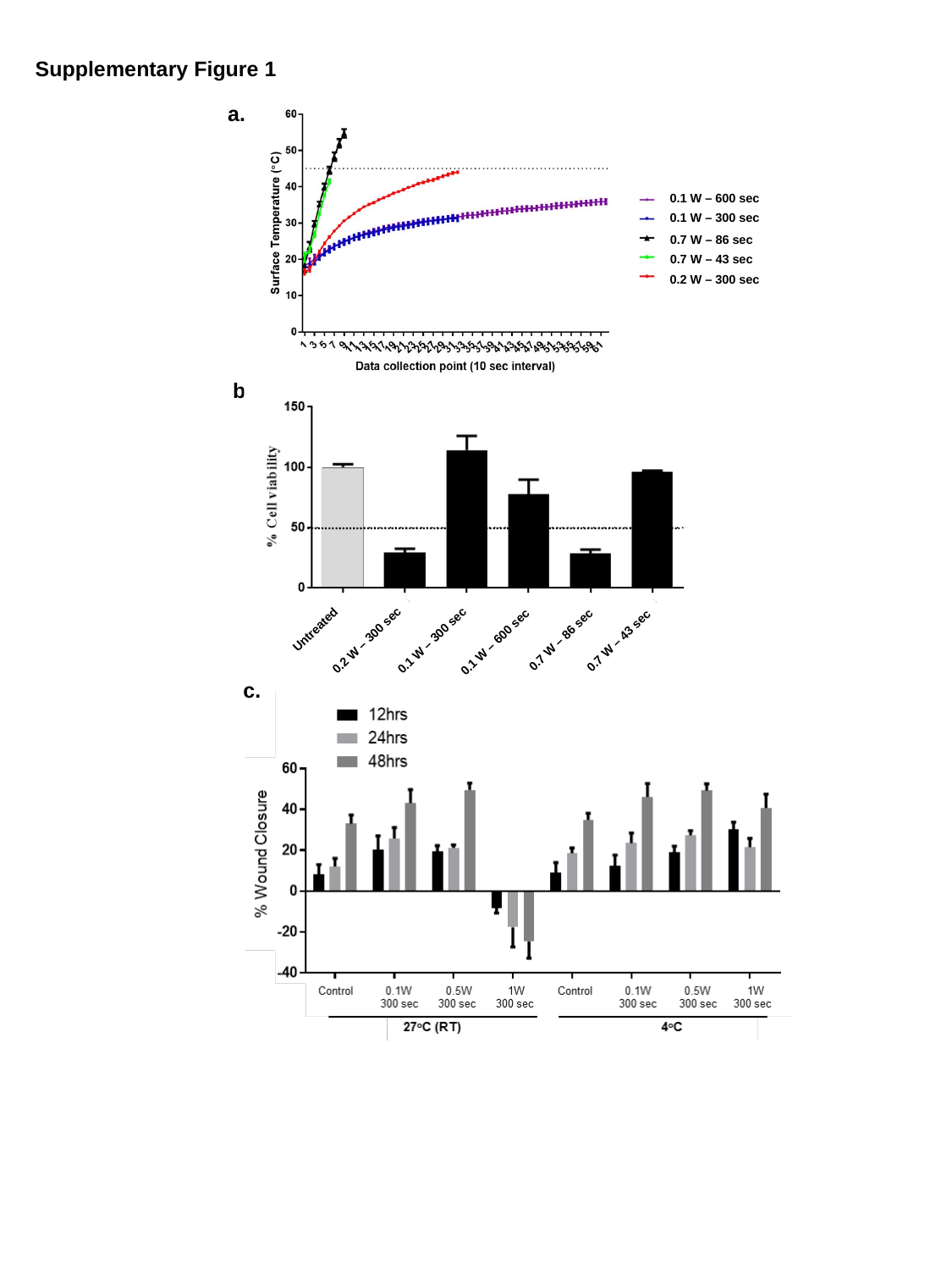

Supplementary Figure 1
a.
0.1 W – 600 sec
0.1 W – 300 sec
0.7 W – 86 sec
0.7 W – 43 sec
0.2 W – 300 sec
b.
0.2 W – 300 sec
0.1 W – 300 sec
Untreated
0.1 W – 600 sec
0.7 W – 86 sec
0.7 W – 43 sec
c.

## Slide 2
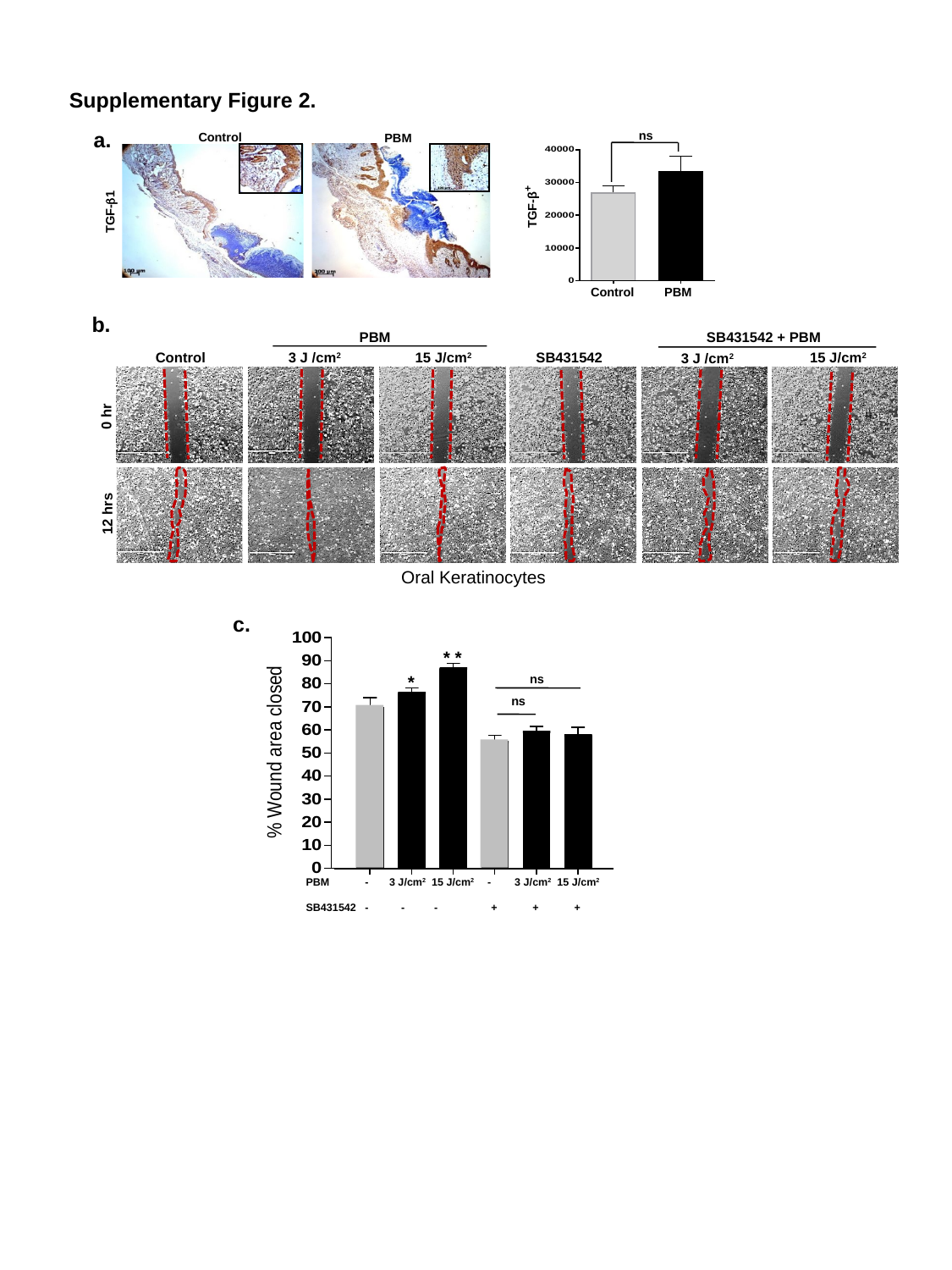

Supplementary Figure 2.
a.
ns
Control
PBM
TGF-β+
TGF-β1
Control
PBM
b.
PBM
SB431542 + PBM
Control
3 J /cm2
15 J/cm2
SB431542
15 J/cm2
3 J /cm2
0 hr
12 hrs
Oral Keratinocytes
c.
* *
ns
ns
PBM - 3 J/cm2 15 J/cm2 - 3 J/cm2 15 J/cm2
SB431542 - - - + + +
*

## Slide 3
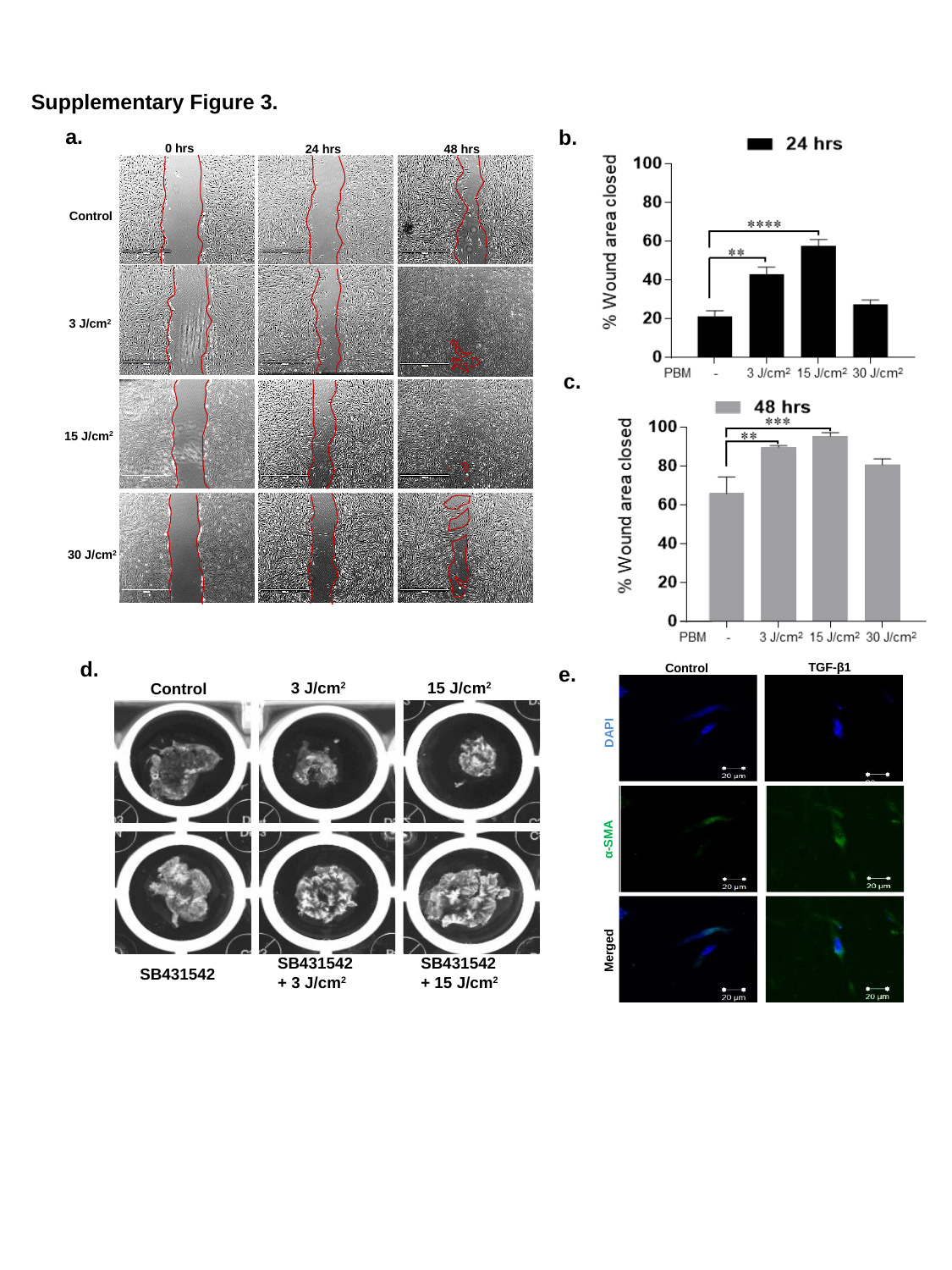

Supplementary Figure 3.
a.
b.
0 hrs
24 hrs
48 hrs
Control
3 J/cm2
15 J/cm2
30 J/cm2
c.
d.
TGF-β1
Control
DAPI
α-SMA
Merged
e.
3 J/cm2
15 J/cm2
Control
SB431542
+ 3 J/cm2
SB431542
+ 15 J/cm2
SB431542

## Slide 4
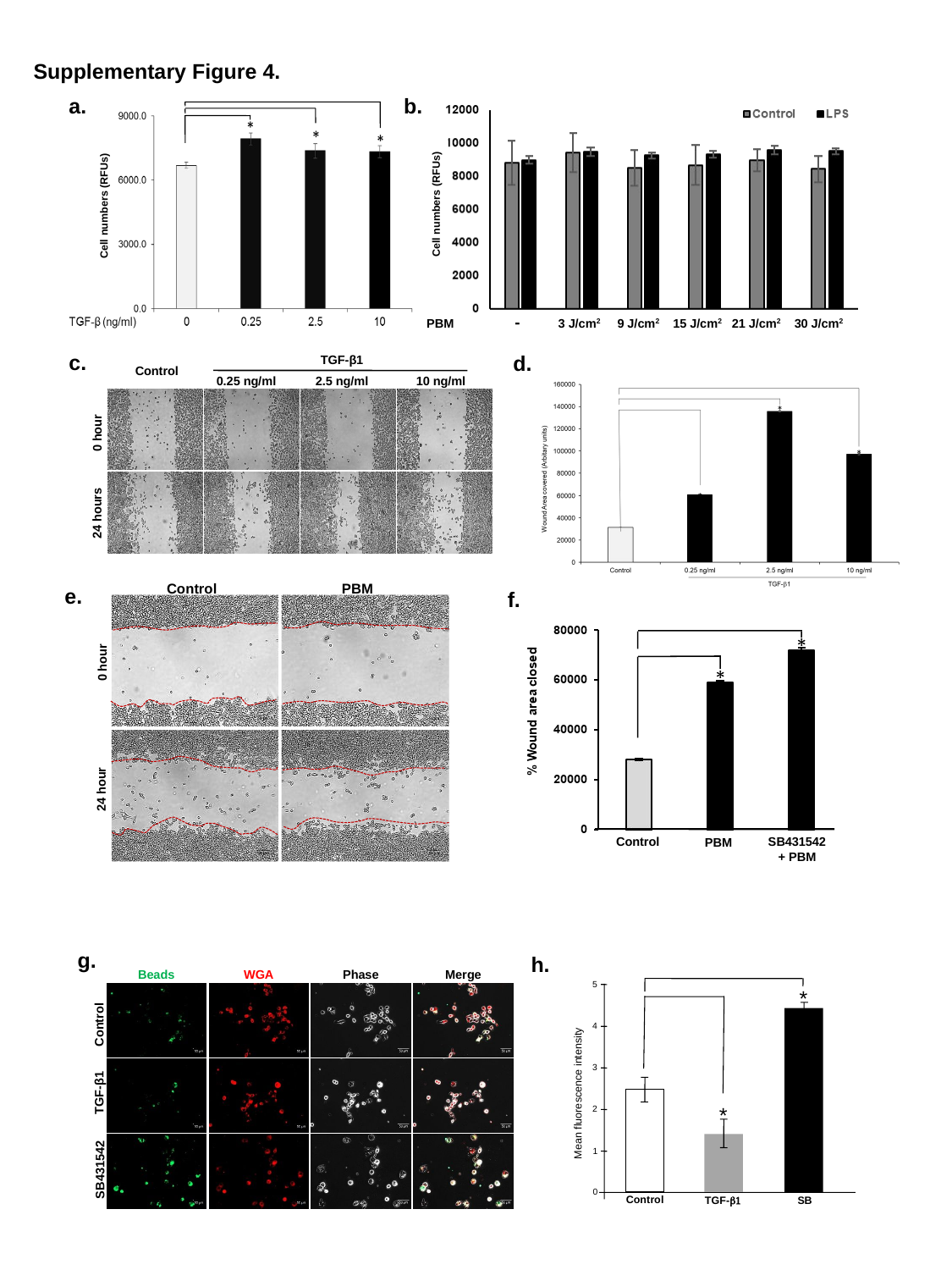

Supplementary Figure 4.
a.
b.
Cell numbers (RFUs)
Cell numbers (RFUs)
PBM - 3 J/cm2 9 J/cm2 15 J/cm2 21 J/cm2 30 J/cm2
c.
d.
TGF-β1
2.5 ng/ml
0.25 ng/ml
10 ng/ml
Control
0 hour
24 hours
Control
PBM
0 hour
24 hour
e.
f.
SB431542 + PBM
Control
PBM
g.
h.
Beads
WGA
Phase
Merge
Control
TGF-β1
SB431542
5
*
4
3
Mean fluorescence intensity
*
2
1
0
Control
TGF-
SB

## Slide 5
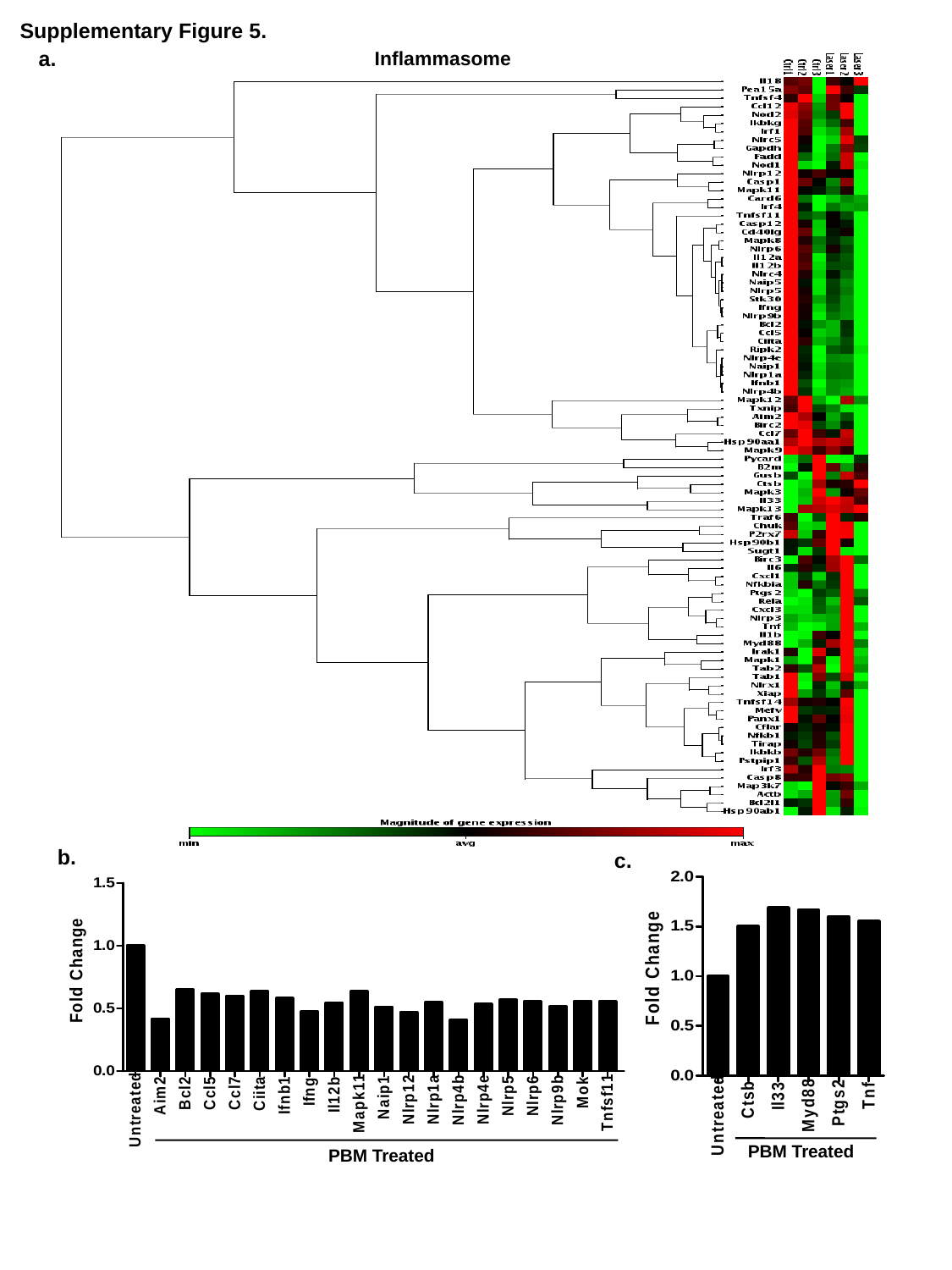

Supplementary Figure 5.
a.
Inflammasome
b.
c.
PBM Treated
PBM Treated

## Slide 6
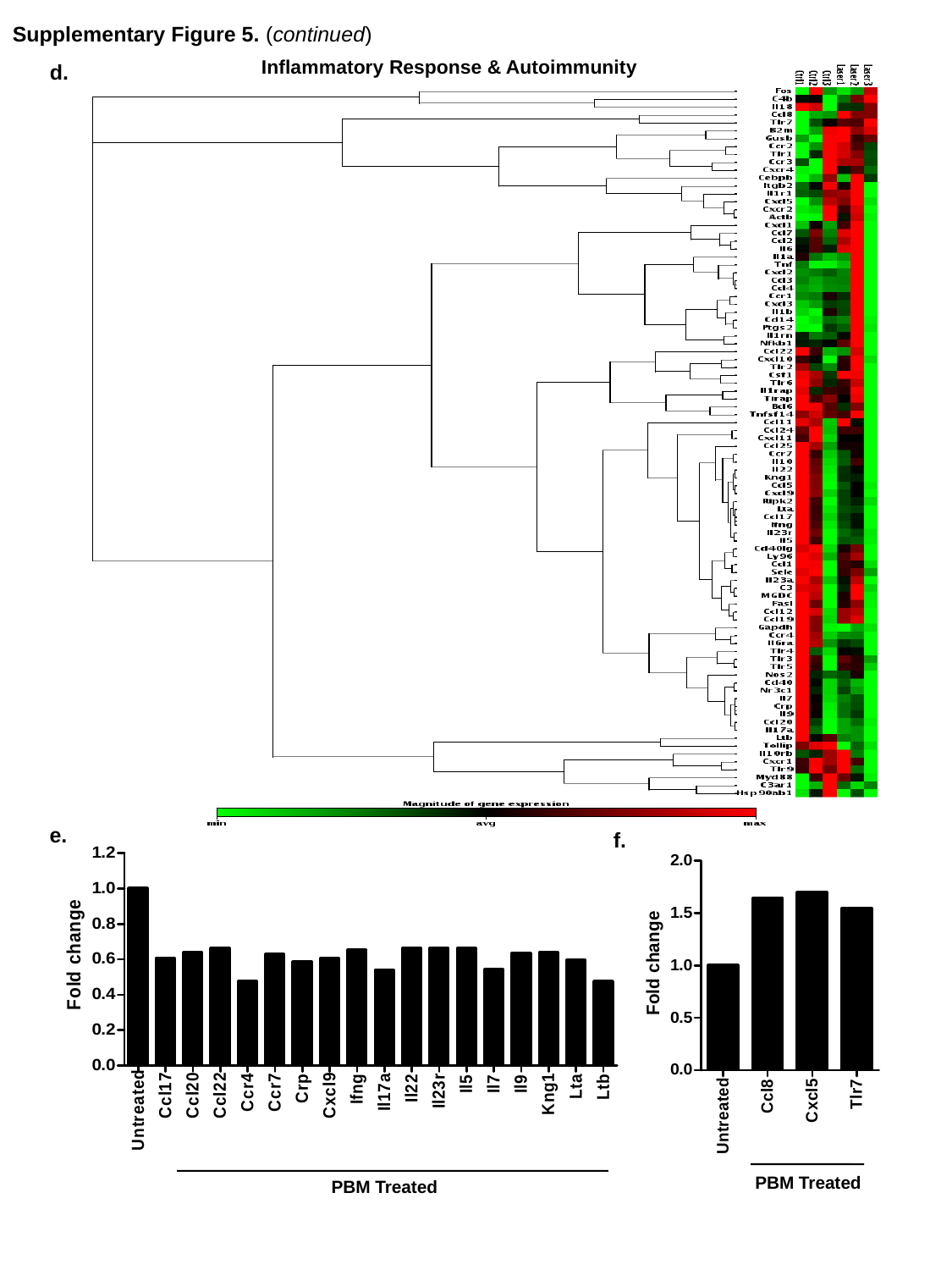

Supplementary Figure 5. (continued)
Inflammatory Response & Autoimmunity
d.
e.
f.
PBM Treated
PBM Treated

## Slide 7
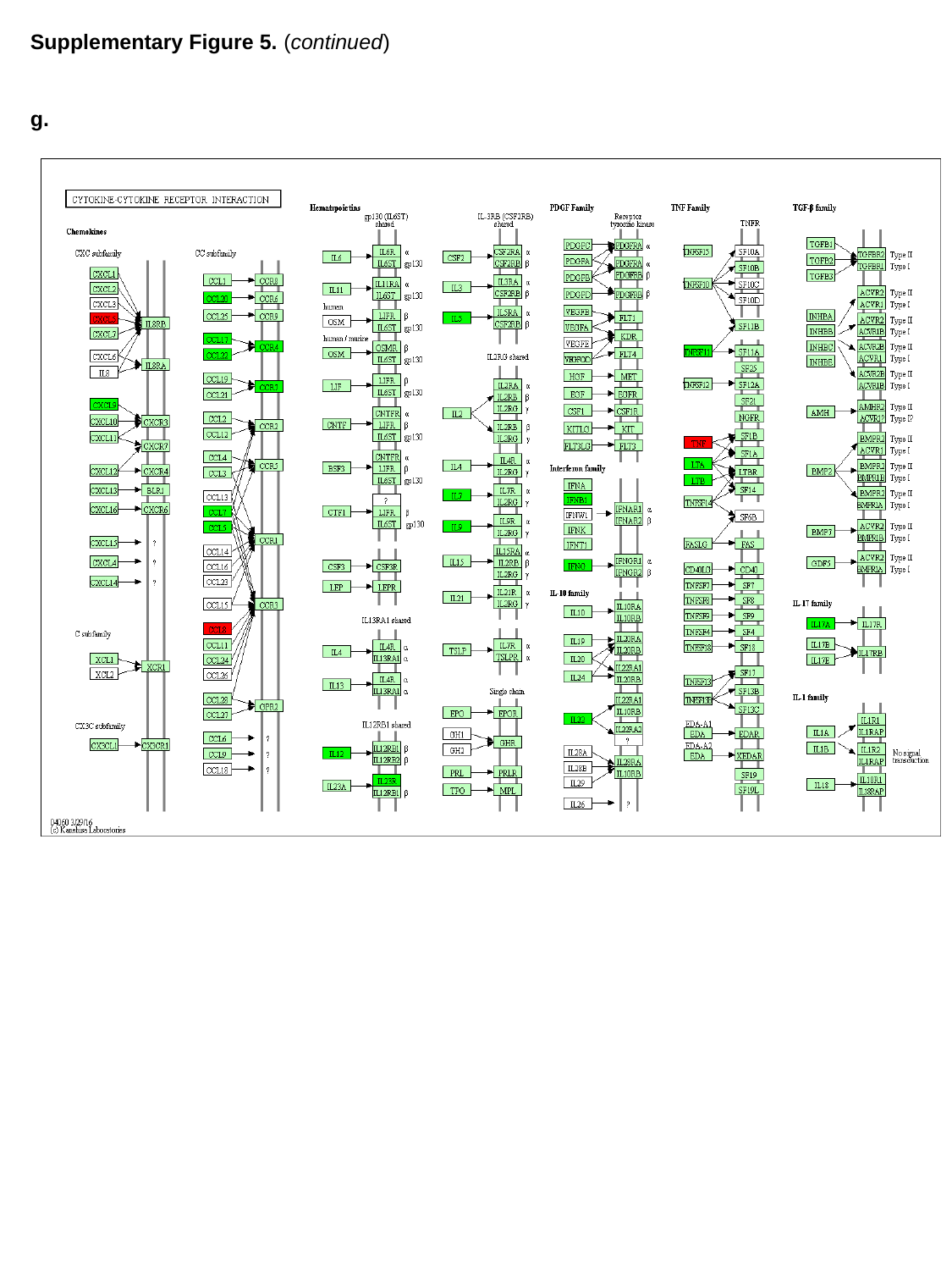

Supplementary Figure 5. (continued)
g.

## Slide 8
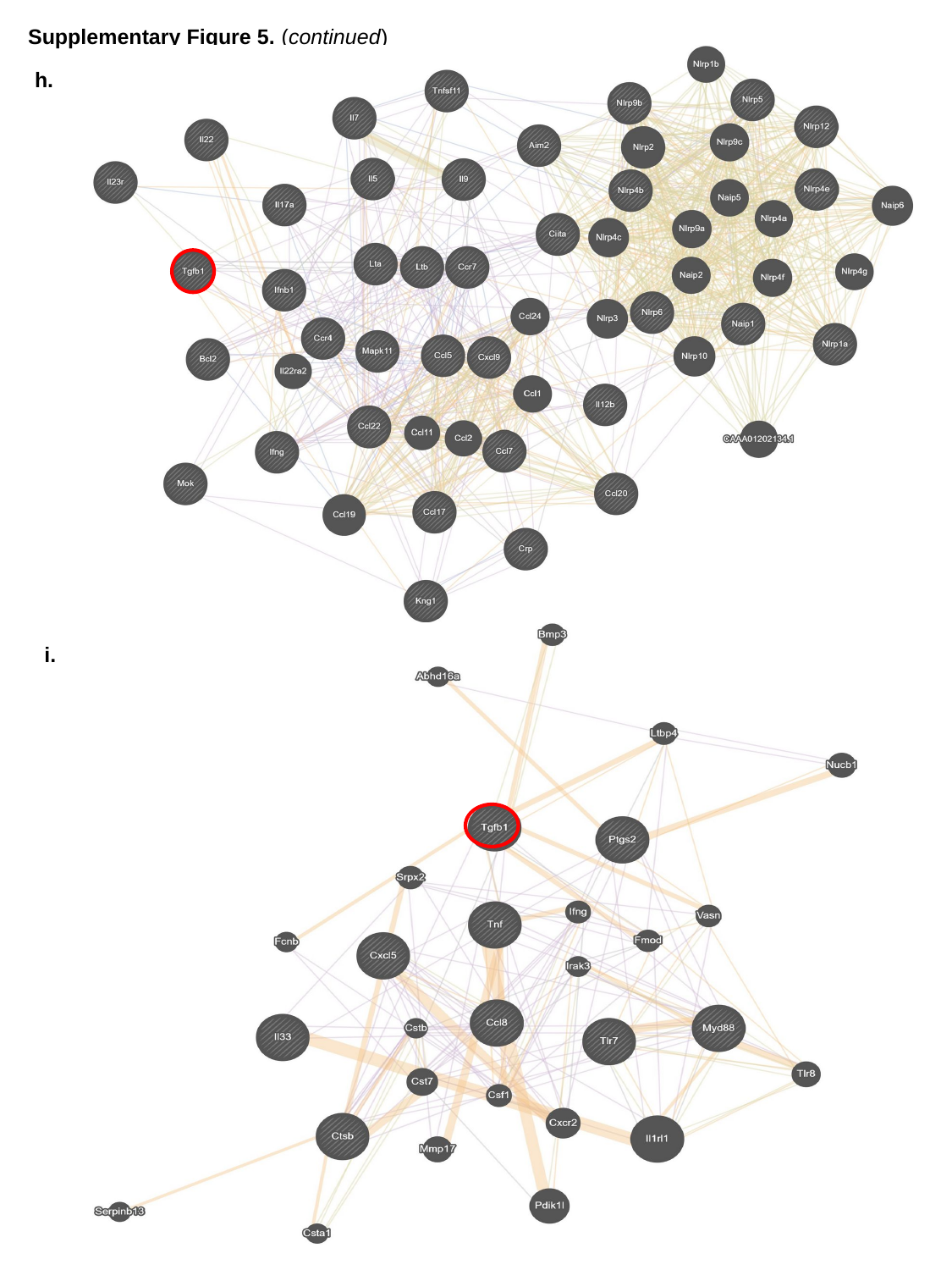

Supplementary Figure 5. (continued)
h.
i.

## Slide 9
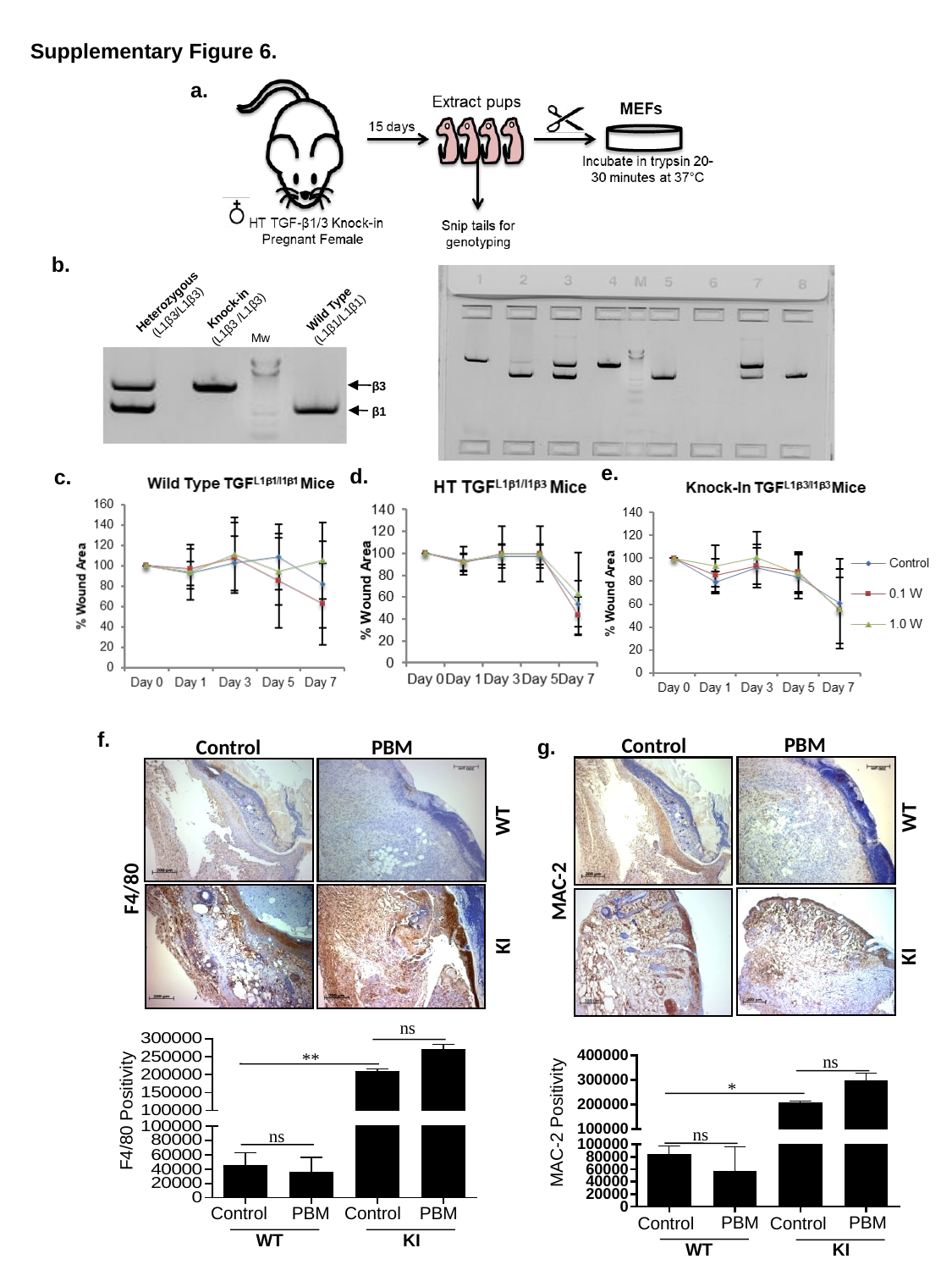

Supplementary Figure 6.
a.
b.
Heterozygous
(L1β3/L1β3)
Knock-in
(L1β3 /L1β3)
Wild Type
(L1β1/L1β1)
Mw
β3
β1
e.
d.
c.
f.
PBM
Control
WT
MAC-2
KI
PBM
Control
WT
F4/80
KI
g.
ns
**
F4/80 Positivity
ns
PBM
PBM
Control
Control
WT
KI
ns
*
MAC-2 Positivity
ns
PBM
PBM
Control
Control
WT
KI

## Slide 10
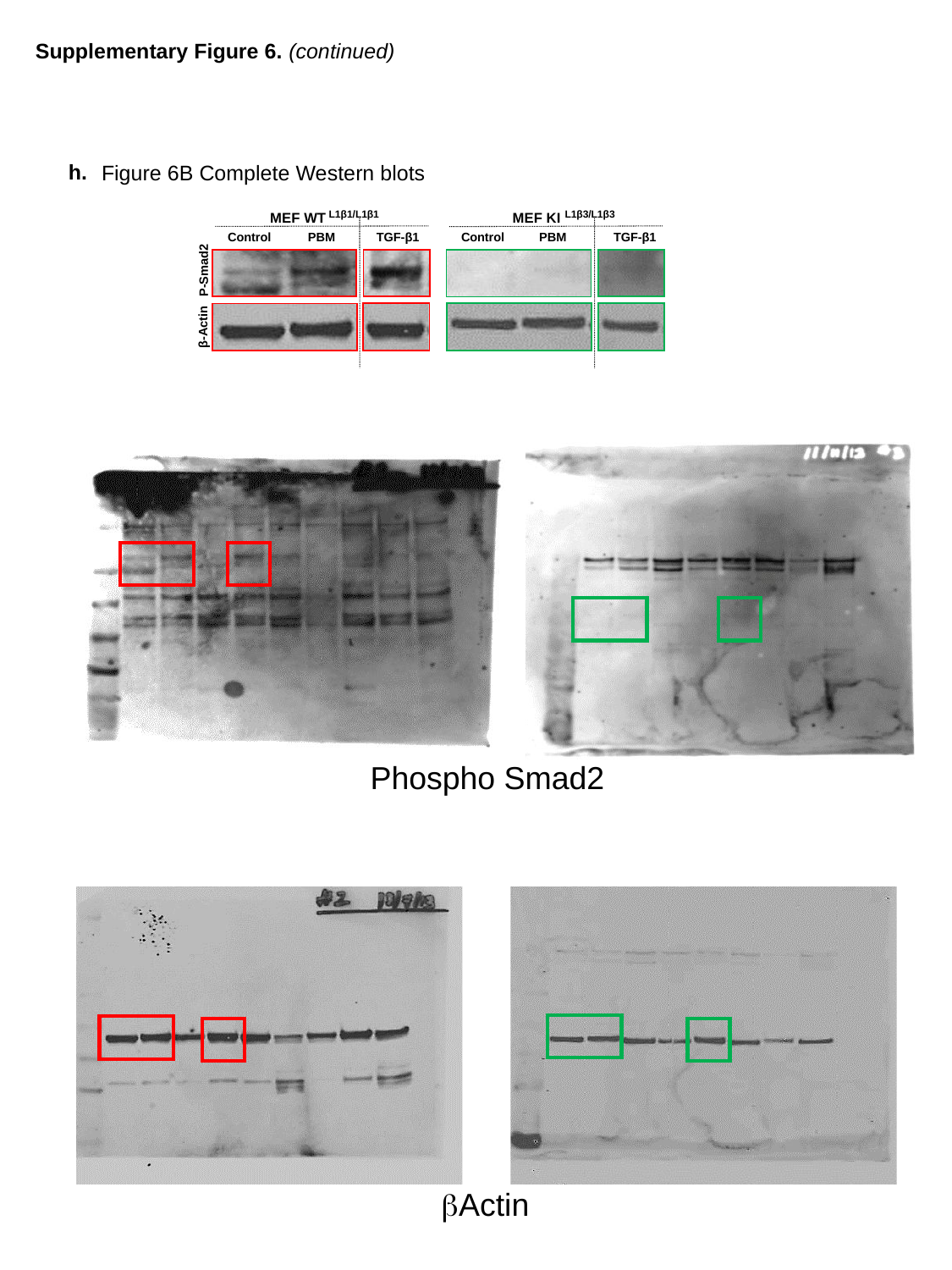

Supplementary Figure 6. (continued)
h.
Figure 6B Complete Western blots
MEF WT L1β1/L1β1
MEF KI L1β3/L1β3
Control
PBM
TGF-β1
Control
PBM
TGF-β1
P-Smad2
β-Actin
Phospho Smad2
βActin

## Slide 11
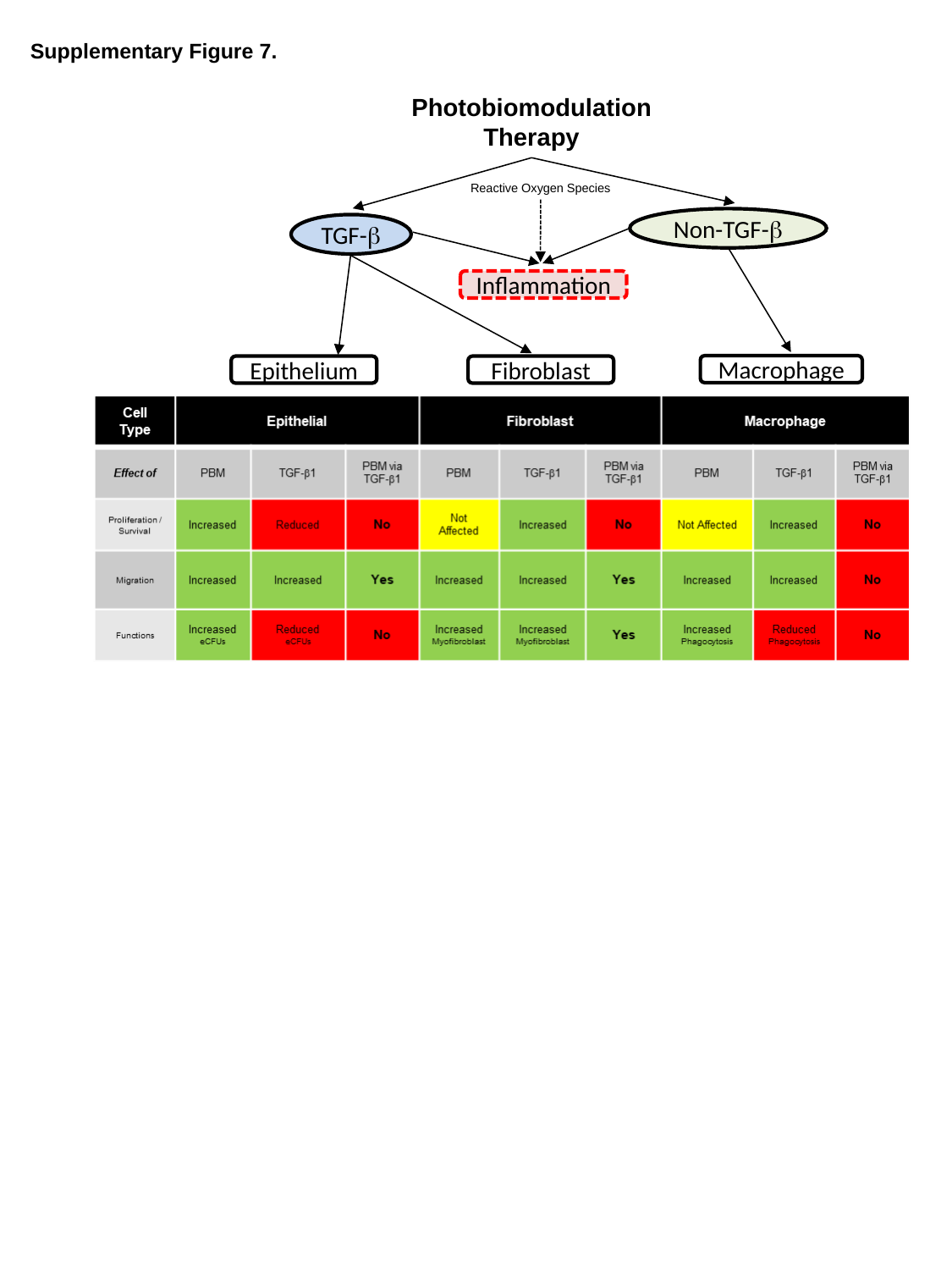

Supplementary Figure 7.
Photobiomodulation Therapy
Reactive Oxygen Species
Non-TGF-β
TGF-β
Inflammation
Macrophage
Epithelium
Fibroblast
